# Iron-mediated aggregation and toxicity in a novel neuronal cell culture model with inducible alpha-synuclein expression

**DOI:** 10.1038/s41598-019-45298-6

**Published:** 2019-06-24

**Authors:** Martin Bartels, Daniel Weckbecker, Peer-Hendrik Kuhn, Sergey Ryazanov, Andrei Leonov, Christian Griesinger, Stefan F. Lichtenthaler, Kai Bötzel, Armin Giese

**Affiliations:** 1 Center for Neuropathology and Prion Research, Ludwig-Maximilians-University, Munich, Germany; 2 Department of Neurology, Klinikum der Universität München, Munich, Germany; 3 MODAG GmbH, Wendelsheim, Germany; 4 Institute of Pathology, TUM School of Medicine, Technical University of Munich, Munich, Germany; 5 Center for Nanoscale Microscopy and Molecular Physiology of the Brain, Georg-August-University Göttingen, 37073 Göttingen, Germany; 6 Department of NMR-based Structural Biology, Max Planck Institute for Biophysical Chemistry, 37077 Göttingen, Germany; 7 German Center for Neurodegenerative Diseases (DZNE), and Munich Cluster for Systems Neurology (SyNergy), Munich, Germany; 8 Neuroproteomics, School of Medicine, Klinikum rechts der Isar, and Institute for Advanced Science, Technical University of Munich, 81675 Munich, Germany

**Keywords:** Cellular imaging, High-throughput screening, Cellular neuroscience, Parkinson's disease

## Abstract

Parkinson’s disease (PD) represents an increasing problem in society. The oligomerization of alpha-synuclein (αSyn) is a suggested key event in its pathogenesis, yet the pathological modes of action remain to be fully elucidated. To identify potential disease-modifying therapeutics and to study αSyn-mediated toxic mechanisms, we established cell lines with inducible overexpression of different αSyn constructs: αSyn, αSyn coupled to the fluorescence protein Venus (αSyn-Venus), and αSyn coupled to the N-terminal or C-terminal part of Venus (V1S and SV2, respectively) for a bimolecular fluorescence complementation assay (BiFC). Inducibility was achieved by applying modified GAL4-UAS or Cre-loxP systems and addition of tebufenozide or 4-OH-tamoxifen, respectively. Expression constructs were stably integrated into the host genome of H4 neuroglioma cells by lentiviral transduction. We here demonstrate a detailed investigation of the expression characteristics of inducible H4 cells showing low background expression and high inducibility. We observed increased protein load and aggregation of αSyn upon incubation with DMSO and FeCl_3_ along with an increase in cytotoxicity. In summary, we present a system for the creation of inducibly αSyn-overexpressing cell lines holding high potential for the screening for modulators of αSyn aggregation and αSyn-mediated toxicity.

## Introduction

Parkinson’s disease (PD) represents an increasing problem in society. The loss of dopaminergic neurons in the substantia nigra (SN) and Lewy bodies or Lewy neurites in some of the remaining neurons – with αSyn occurring as highly ordered amyloid-type fibril as principal component – represent the histopathological hallmarks of PD^1–3^, yet cell loss and presence of Lewy bodies are not correlated^4^.

To date, several lines of evidence point towards small αSyn oligomers as driver of toxicity, and the small molecule modulator of αSyn oligomerization 3-(1,3-Benzodioxol-5-yl)-5-(3-bromophenyl)-1*H*-pyrazole shows beneficial effects on pathology in *in vivo* models of synucleinopathies^5^, yet – to date – the distinct toxic αSyn species and the pathological mechanisms are not completely understood^6^. It has also been suggested and discussed critically that αSyn may exist mainly as physiological tetramer^7,8^ and that the disruption of tetrameric αSyn to unfolded monomers represents the starting point for pathological αSyn aggregation^9^. Interestingly, the overexpression of αSyn appears to be sufficient to induce pathological effects, since duplication or triplication of the *SNCA* gene cause parkinsonian symptoms with the age of onset and severity of symptoms correlating with the gene copy number^10–12^. Additionally, modulating SNCA transcription by targeting the β2-adrenoreceptor (β2AR) using salbutamol is associated with reduced risk of developing PD^13^. In several epidemiological studies environmental risk factors for the development of PD have been shown to influence αSyn aggregation^14^, including the exposure to heavy metals and iron^15^.

So far, formation, modulation, and toxicity of αSyn oligomers have mainly been studied in cell models in which the overexpression of αSyn is induced by transient transfection^16^ or viral transduction^17^ or in cell models with stable insertion and constitutive overexpression^18,19^. Both strategies hold several drawbacks: (a) when using transient transfection or viral transduction, the fraction of transgene expressing cells and the strength of overexpression are subject to great inter-experimental variation. Moreover, individual experiments are rather time-consuming and expensive. Additionally, the initiation of expression cannot be defined accurately, and for several cell lines transient transfection is very inefficient. (b) Constitutive overexpression of αSyn on the other hand enables the investigation of αSyn oligomers only in steady state but not the investigation of *de novo* oligomer formation. This hinders the identification of compounds which prevent αSyn aggregation but are not capable of degrading or dissociating preformed αSyn aggregates leading to false-negative results in drug screening. Moreover, the constitutive overexpression of αSyn and the resulting αSyn-mediated toxicity may result in selection for cells that are resistant to αSyn-mediated toxicity. This might interfere with the investigation of potential toxic effects.

**Figure 1:**
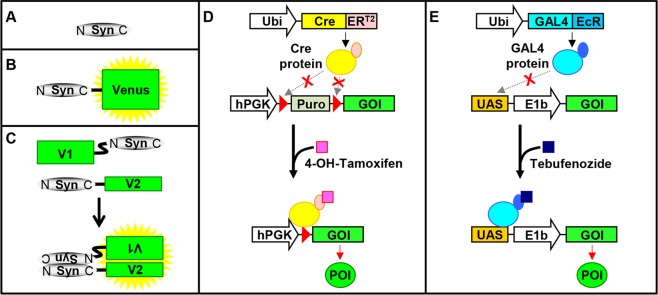
Induction of transgene expression in H4 cells. Overview of αSyn constructs and induction of their overexpression. (**A**) αSyn-140 (S). (**B**) αSyn-140 coupled to the fluorescence protein Venus (SV). (**C**) αSyn-140 coupled to the N-terminal (V1) or C-terminal (V2) part of Venus. Aggregation of αSyn results in fluorescence due to complementation of the Venus fragments. (**D**) Overexpression in the Cre_ER^T2^-loxP system (CE^T2^) is induced by 4-OH-tamoxifen. (**E**) Overexpression in the GAL4_EcR-UAS system (GE) is induced by tebufenozide.

For these reasons, we here present a system for the fast and easy creation of cell lines with inducible overexpression of different proteins, namely αSyn-140 (S) (Fig. 1A) which is the most abundant splice-variant of αSyn in humans, the YFP variant Venus (V)^20^, αSyn-140 coupled to Venus (SV) (Fig. 1B), the N-terminal part of Venus coupled to αSyn-140 (V1S), and αSyn-140 coupled to the C-terminal part of Venus (SV2), where the co-expression of V1S and SV2 can be used for a bimolecular fluorescence complementation assay (BiFC) (Fig. 1C)^19,21^.

Overexpression relied on the Cre_ER^T2^-loxP (CE^T2^) system^22,23^ (Fig. 1D) or the GAL4_EcR-UAS (GE)^24^ system (Fig. 1E). Both systems for inducible expression were successfully inserted into H4 cells mediated by lentiviral transduction^25^. Since the H4 cell lines relying on the GE system (H4_GE cells) showed higher transgene induction than the H4 cell lines relying on the CE^T2^ system (H4_CE^T2^ cells), the expression characteristics of the tebufenozide-dependent H4_GE cells were analysed in detail. Using this cell model we further found that incubation with DMSO and ferric iron led to an increase in fluorescence intensity in the BiFC assay indicating αSyn oligomer formation which was confirmed by sucrose gradient centrifugation. Increased aggregation was accompanied by an increase in cytotoxicity as demonstrated by quantification of condensed nuclei in both H4_GE cells expressing V1S + SV2 and untagged αSyn, further validating the value of our model for studying risk factors of PD development, analysing clearance mechanisms, or screening for therapeutic compounds.

## Results

### Transgene expression after lentiviral transduction

After production and purification of the required lentiviruses to prepare stable cell lines we established an optimized transduction protocol to obtain as many lentivirally transduced H4 cells as possible (Supplementary Fig. 1). Following protocol D, we obtained 98% of Venus-positive cells and the fraction of positive cells remained stable for at least 18 days after transduction. 23 days after transduction we observed a decrease in the fraction of positive cells (95%) which was not significant (p = 0.36) (Supplementary Fig. 2).

As a consequence, stable cell lines were created following protocol D. This resulted in the following cell lines with inducible expression based on the GE system: H4_GE-S (inducible overexpression of αSyn), H4_mC_GE-S (constitutive expression of mCherry coupled to a nuclear localization sequence (NLS) resulting in red fluorescence in the nucleus of positively transduced cells and inducible overexpression of αSyn), H4_GE-V (inducible overexpression of Venus), H4_GE-SV (inducible overexpression of αSyn coupled to Venus), and H4_GE-V1S + SV2 (inducible overexpression of V1S and SV2).

The following cell lines with inducible expression based on the CE^T2^ system were created: H4_CE^T2^-S (inducible overexpression of αSyn), H4_CE^T2^-V (inducible overexpression of Venus), H4_CE^T2^-SV (inducible overexpression of αSyn coupled to Venus), and H4_CE^T2^-V1S + SV2 (inducible overexpression of V1S and SV2) (see Fig. 1).

### Superior induction of transgene expression in H4_GE cells compared to H4_CE^T2^ cells

In order to test the generated H4_GE cell lines, we incubated them with 10 µM tebufenozide or 0.1% DMSO as control for 48 h and analysed the cells using the Opera^®^ reader. To determine the factorial increase in transgene expression upon incubation with tebufenozide, the background signal from H4 cells was subtracted from the signal of the corresponding inducible H4_GE cells after treatment with tebufenozide or DMSO.

**Figure 2:**
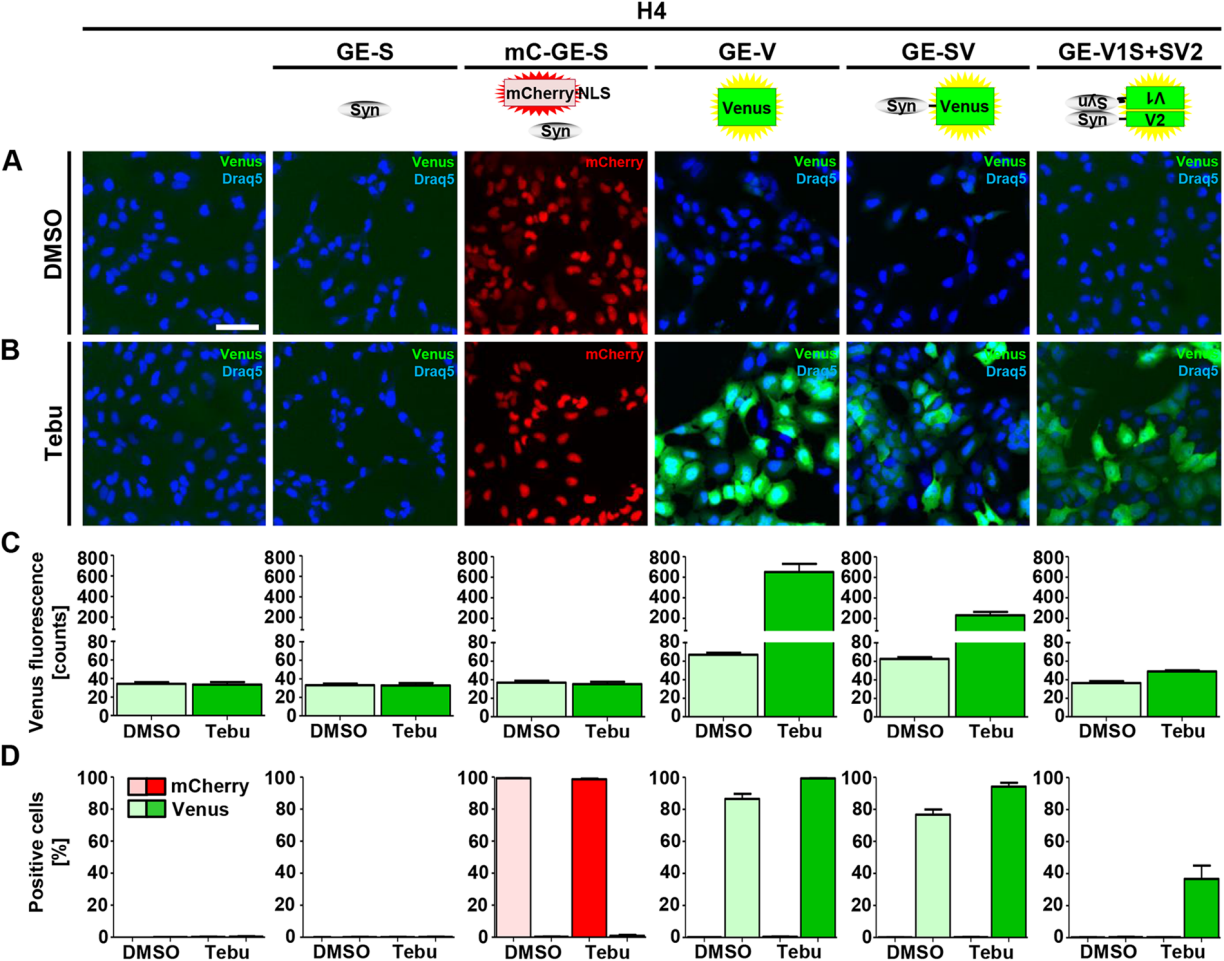
Induction of transgene expression in H4_GE cells. The generated H4_GE cell lines were incubated with 0.1% DMSO or 10 µM tebufenozide for 48 h and imaged using the Opera^®^ system. Quantification was performed using the Acapella^®^ software. Nuclei were stained with Draq5. (**A**) Representative fluorescence images of cells incubated with 0.1% DMSO. Scale bar is 100 µm and valid for all panels in A and B. (**B**) Representative fluorescence images of cells incubated with 10 µM tebufenozide. (**C**) Quantification of the mean cellular Venus fluorescence intensity of H4 cells. (**D**) Quantification of the fraction of Venus or mCherry-positive H4 cells. Bars in C and D show mean of 3 independent experiments; error bars show SEM.

As expected, we observed no green fluorescence in H4 cells, H4_GE-S cells, or H4_mC_GE-S cells independent of treatment with DMSO (Fig. 2A,C) or tebufenozide (Fig. 2B,C) and the fraction of Venus-positive cells was below 1%. Approximately 99% of H4_mC_GE-S cells were mCherry-positive independent of tebufenozide or DMSO treatment, while the fraction of mCherry-positive cells was 0% in all other cell lines as expected. Thus, 99% of H4_mC_GE-S cells were transduced successfully with the mCherry-NLS-UAS-αSyn receiver virus (V120) (Fig. 2D).

Following incubation with tebufenozide, fluorescence intensity increased considerably from 33 to 616 counts in H4_GE-V cells, from 29 to 198 counts in H4_GE-SV cells and from approximately 2.6 to 15.1 counts in H4_GE-V1S + SV2 cells, suggesting an increase in transgene expression following tebufenozide treatment by the factor 18.7, 6.8, and 5.8, respectively (Fig. 2B,C). All cells – including Venus-negative cells – were included in the evaluation of the mean cellular Venus fluorescence of the H4_GE-V1S + SV2 cells.

For H4_GE-V and H4_GE-SV cells we observed increased background fluorescence upon treatment with DMSO compared to untreated H4 cells, whereas background fluorescence was in the range of unmodified H4 cells for DMSO-treated H4_GE-V1S + SV2 cells (Fig. 2A,C).

H4_CE^T2^ cell lines were incubated with 10 µM 4-OH-tamoxifen (since this yielded the highest fluorescence intensity without obvious adverse effects on cell shape and cell survival (Supplementary Fig. 2)) or DMSO as control for 48 h and analysed using the Opera^®^ reader. Since we obtained a stronger increase in transgene expression upon treatment with tebufenozide in the H4_GE cells compared to 4-OH-tamoxifen treatment in the H4_CE^T2^ cells (18.6-fold vs. 2.9-fold for Venus, and 6.8-fold vs. 2.4-fold for Syn-Venus, Supplementary Fig. 3), we continued with the GE-based system for the characterization of expression kinetics.

### Expression characteristics of H4_GE Cells – cellular fluorescence

In order to characterize the created H4_GE cell lines regarding their transgene expression over time and depending on tebufenozide concentration, all cell lines were incubated for up to 8 days post induction (DPI) with different tebufenozide concentrations ranging from 100 pM to 100 µM or DMSO as control, respectively.

**Figure 3:**
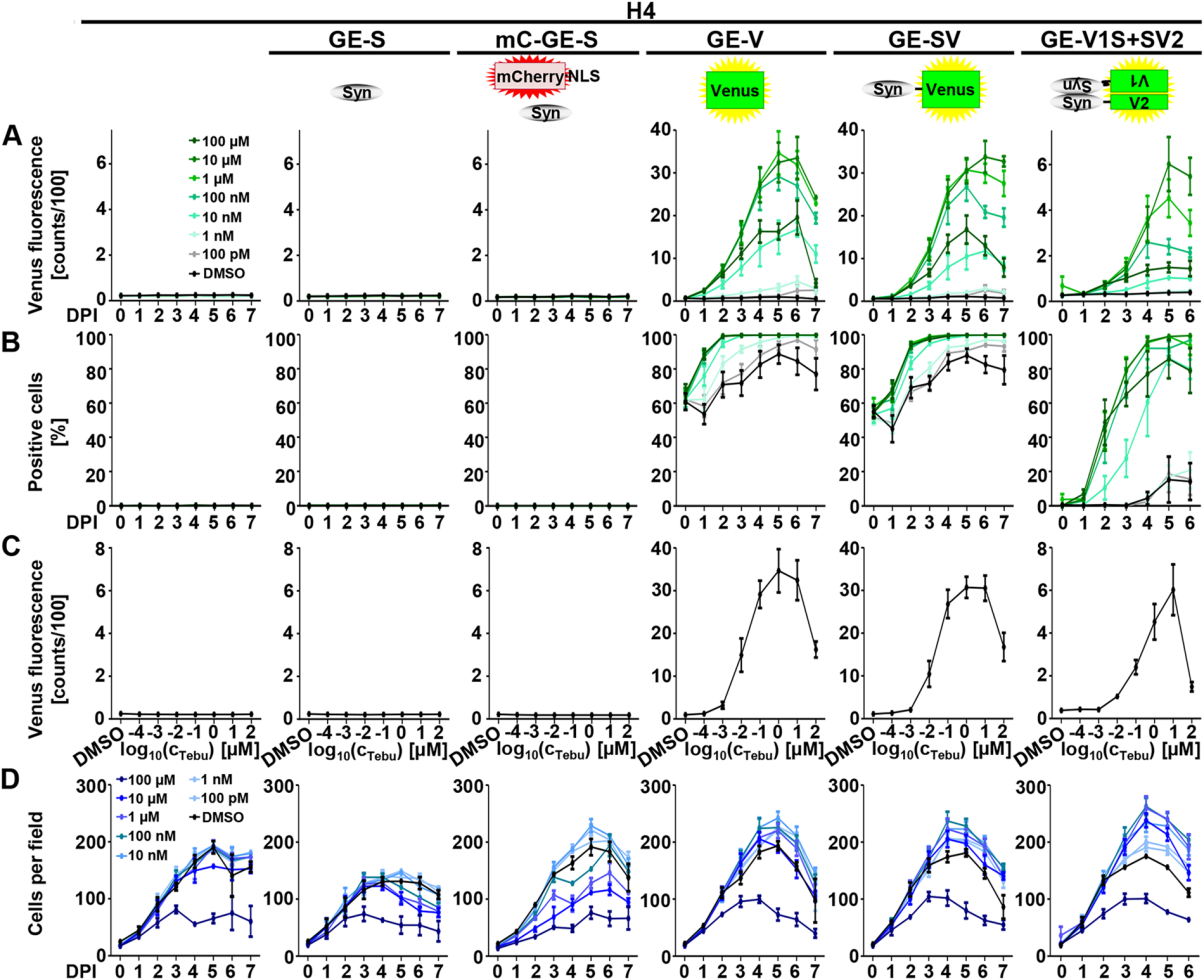
Expression characteristics of H4_GE cells – cellular fluorescence. H4 cell lines were incubated for up to 8 days with DMSO or with different tebufenozide concentrations ranging from 100 pM to 100 µM and imaged using the Opera^®^ system. Quantification was performed using the Acapella^®^ software. (**A**) Quantification of the mean cellular Venus fluorescence intensity of H4 cells upon incubation with DMSO or different concentrations of tebufenozide (color-coded). (**B**) Quantification of the fraction of Venus-positive H4 cells. (**C**) Quantification of the mean cellular Venus fluorescence intensity of H4 cells in dependence of the tebufenozide concentration using the dataset from A) at DPI 5. (**D**) Quantification of the number of cells per image field. Data points show mean of 3–6 independent experiments; error bars show SEM.

No increase in mean cellular Venus fluorescence was observed in H4, H4_GE-S, or H4_mC_GE-S cells (Fig. 3A). In line with this, the fraction of positive cells remained 0% for all time points and tebufenozide concentrations in H4, H4_GE-S, or H4_mC_GE-S cells (Fig. 3B). Accordingly, no influence of tebufenozide concentration on fluorescence intensity could be observed in these cell lines (color-coded in Fig. 3A,B, and visualized for day 5 in C).

On the contrary, the fluorescence signal increased in a time- and tebufenozide concentration-dependent manner from day 0 on in H4_GE-V, H4_GE-SV, and H4_GE-V1S + SV2 cells (Fig. 3A).

In H4_GE-V cells, maximum fluorescence was obtained upon incubation with 1 µM tebufenozide on DPI 5 (Fig. 3A,C) with an increase from approximately 37.9 counts (for DMSO-treated cells on DPI 0) to 3,439.0 counts (all values are indicated after subtraction of background signal), equivalent to a 90.8-fold increase in fluorescence intensity. The fraction of positive cells increased from approximately 63% on DPI 0 (due to background fluorescence) to 100% on DPI 3 to 7 for cells treated with 1 µM or 10 µM tebufenozide, while a maximum of about 89% of DMSO-treated cells were considered Venus-positive on DPI 5.

For H4_GE-SV cells, maximum fluorescence was obtained upon incubation with 10 µM tebufenozide on DPI 6 (Fig. 3A) with an increase from approximately 34.6 counts (for DMSO-treated cells at day 0) to 3,350.6 counts, equivalent to a 96.9-fold increase in fluorescence intensity. The fraction of positive cells increased from approximately 55% on DPI 0 (due to background fluorescence) to 100% on DPI 4 to 7 for cells treated with 10 µM tebufenozide, while a maximum of approximately 88% of DMSO-treated cells was Venus-positive on DPI 5 (Fig. 3B).

In H4_GE-V1S + SV2 cells fluorescence intensity increased more slowly than in H4_GE-V and H4_GE-SV cells and reached its maximum on DPI 6 upon incubation with 10 µM tebufenozide (Fig. 3A) with an increase from approximately 0.4 counts (for DMSO-treated cells at DPI 0) to 520.5 counts, equivalent to an 1,240.7-fold increase in fluorescence intensity following tebufenozide treatment. The fraction of positive cells increased from approximately 1% on DPI 0 to 99% on DPI 5 and 6 for cells treated with 10 µM tebufenozide, while a maximum of approximately 15% of DMSO-treated cells was Venus-positive on DPI 5 (Fig. 3B).

For H4_GE-V, H4_GE-SV, and H4_GE-V1S + SV2 cells, fluorescence intensity increased with increasing tebufenozide concentrations in the range of 100 pM to 10 µM and decreased when cells were incubated with 100 µM tebufenozide (Fig. 3C).

For all cell lines the number of cells increased until DPI 4 or 5. No obvious effect on cell number was observed for all tebufenozide concentrations except for treatment with 100 µM tebufenozide where the number of cells was decreased in all cell lines.

### Expression characteristics of H4_GE cells – protein amount depending on tebufenozide concentration

In order to further characterize the cell lines regarding their transgene expression properties in dependence of the tebufenozide concentration, the cells were incubated for 4 days with different tebufenozide concentrations ranging from 10 nM to 10 µM or with DMSO as control, respectively. After 4 days cells were harvested, lysed and subjected to Western blot analyses.

**Figure 4:**
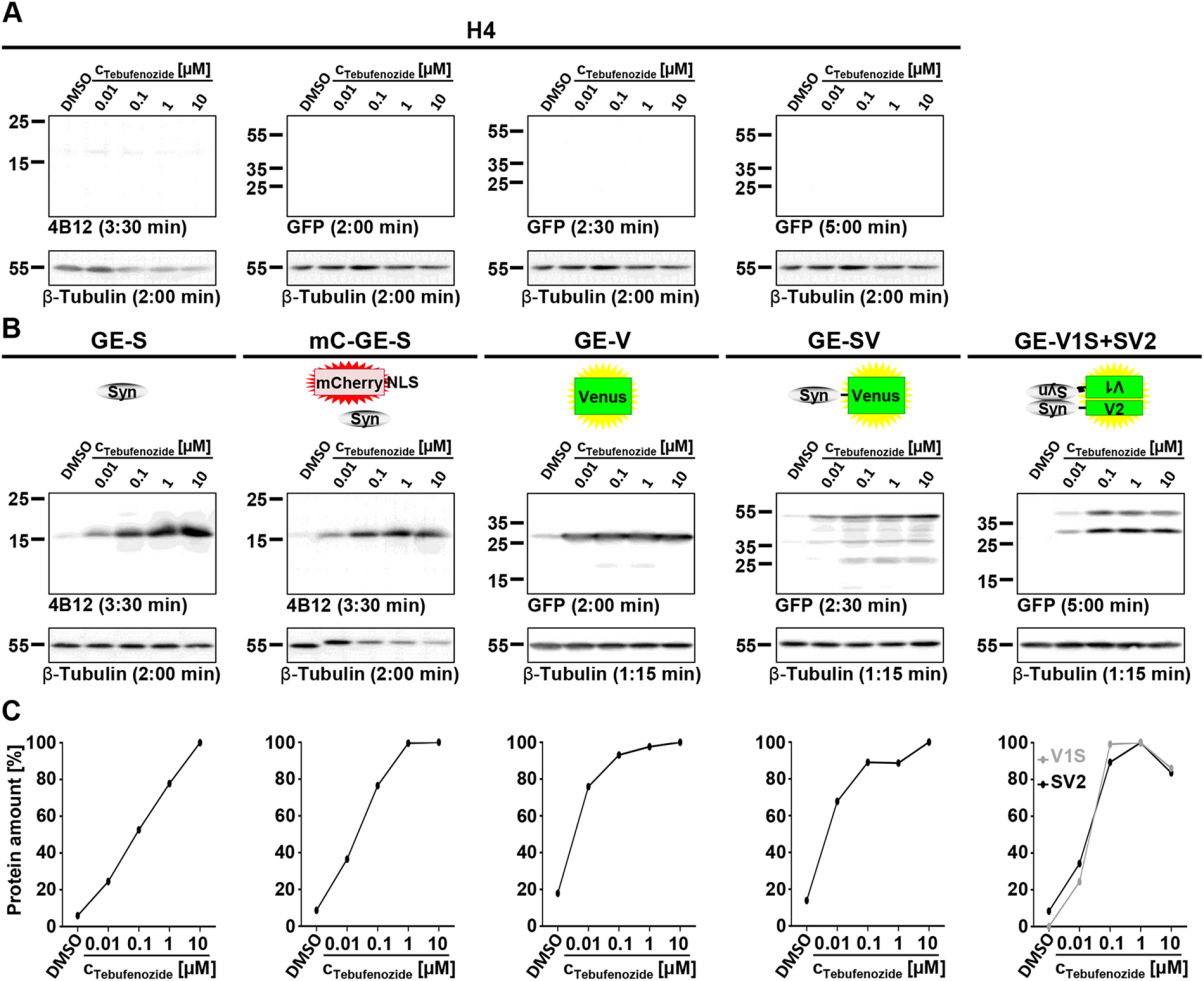
Expression characteristics of H4_GE cells – protein amounts depending on tebufenozide concentration. H4 cell lines were incubated for 4 days with DMSO or different tebufenozide concentrations ranging from 10 nM to 10 µM. For all samples, expression of housekeeper β-tubulin was used as reference. (**A**) Control Western blots of H4 cells were treated like the Western blots of the corresponding samples and showed no expression of αSyn, Venus, SV, V1S, or SV2. (**B**) In all transgene expressing cell lines signal intensity increased with increasing tebufenozide concentration up to 1 µM or 10 µM for inducible transgenes. Full-length Western blots and additional exposures are shown in Supplementary Figs 5, 6, respectively. (**C**) Quantification of Western blot signal intensity was performed using *ImageJ*. Signal intensity of transgenes was normalized to maximum. Data points were derived from the blots shown in A and B.

As expected, we observed no transgene expression in H4 cells (Fig. 4A). For all transgene-expressing cell lines we observed very little background expression upon incubation with DMSO. In all cases, protein amounts increased with increasing tebufenozide concentration up to 1 or 10 µM (Fig. 4B).

Cell lines H4_GE-S, H4_mC_GE-S, H4_GE-V, and H4_GE-SV showed a maximum in transgene expression after incubation with 10 µM tebufenozide, whereas maximum transgene expression in H4_GE-V1S + SV2 was observed upon incubation with 1 µM tebufenozide. According to the quantification of Western blot signal intensity we obtained a maximum increase in transgene expression compared to DMSO by a factor of 16.8 in H4_GE-S, 11.5 in H4_mC_GE-S, 5.6 in H4_GE-V, 7.2 in H4_GE-SV, and 11.9 in H4_GE-V1S + SV2 for SV2 expression. Concerning the expression of V1S in H4_GE-V1S + SV2 the factorial increase in transgene expression could not be evaluated since no background signal for treatment with DMSO was detected.

### Expression characteristics of H4_GE cells – kinetics of protein amount

In order to further characterize the cell lines regarding their expression kinetics according to protein levels, the cells were incubated for up to 5 days with 10 µM tebufenozide since this reached the highest transgene expression in most cell lines (see Figs 3, 4). Cells were harvested, lysed and subjected to Western blot analyses on DPI 0, 1, 2, 3, 4, and 5.

**Figure 5:**
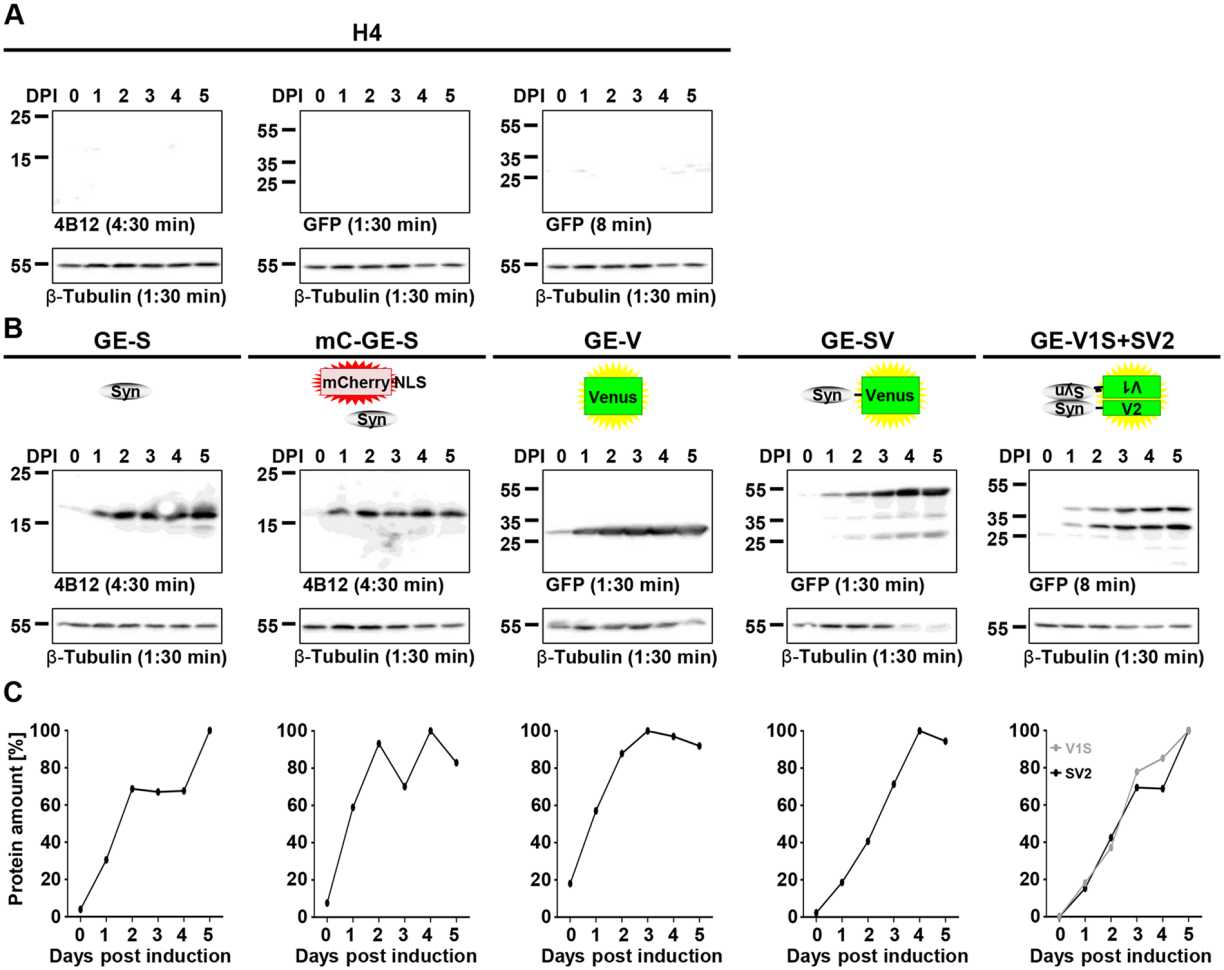
Expression characteristics of H4_GE cells – kinetics of protein amounts. H4 cell lines were incubated with 10 µM tebufenozide for 0 to 5 days. For all samples, expression of housekeeper β-tubulin was used as reference. (**A**) Control Western blots of H4 cells were treated like the Western blots of the corresponding samples and showed no expression of αSyn, Venus, SV, V1S, or SV2. (**B**) In all transgene expressing cell lines signal intensity increased over time for inducible transgenes. Full-length Western blots and additional exposures are shown in Supplementary Figs 7, 8, respectively. (**C**) Quantification of Western blot signal intensity was performed using *ImageJ*. Signal intensity of transgenes was normalized to maximum. Data points were derived from the blots shown in A and B.

As expected, no transgene expression was observed in H4 cells (Fig. 5A). For all transgene expressing cell lines we observed very little background expression in the absence of tebufenozide (DPI 0) and an increase in protein amounts over time (Fig. 5B). According to the quantification of Western blot signal intensity we obtained a maximum increase in protein amount by a factor of 25.1 in H4_GE-S, 13.1 in H4_mC_GE-S, 43.8 in H4_GE-SV, and 5.5 in H4_GE-V. For H4_GE-V1S + SV2 the factorial increase in protein amounts could not be evaluated since no background signal was observed on DPI 0.

### Increased αSyn aggregation and αSyn-Mediated cytotoxicity upon incubation with DMSO and ferric iron

DMSO and ferric iron have been shown to increase aggregation of αSyn *in vitro*^15^. In order to validate our model, we investigated if this effect of DMSO and ferric iron on αSyn aggregation could be reproduced in the H4_GE cell model. For this, H4_GE-V1S + SV2 and H4_GE-V cells were incubated with different combinations of DMSO (ranging from 0.1% to 1.0%) and FeCl_3_ concentrations (ranging from 0 µM to 1 mM) for 3 days. Transgene expression was induced with 10 µM tebufenozide in H4_GE-V1S + SV2 cells and with 10 nM tebufenozide in H4_GE-V cells for three days in order to reach comparable fluorescence intensities. After 3 days, cellular fluorescence intensity was determined using the Opera^®^ setup.

**Figure 6:**
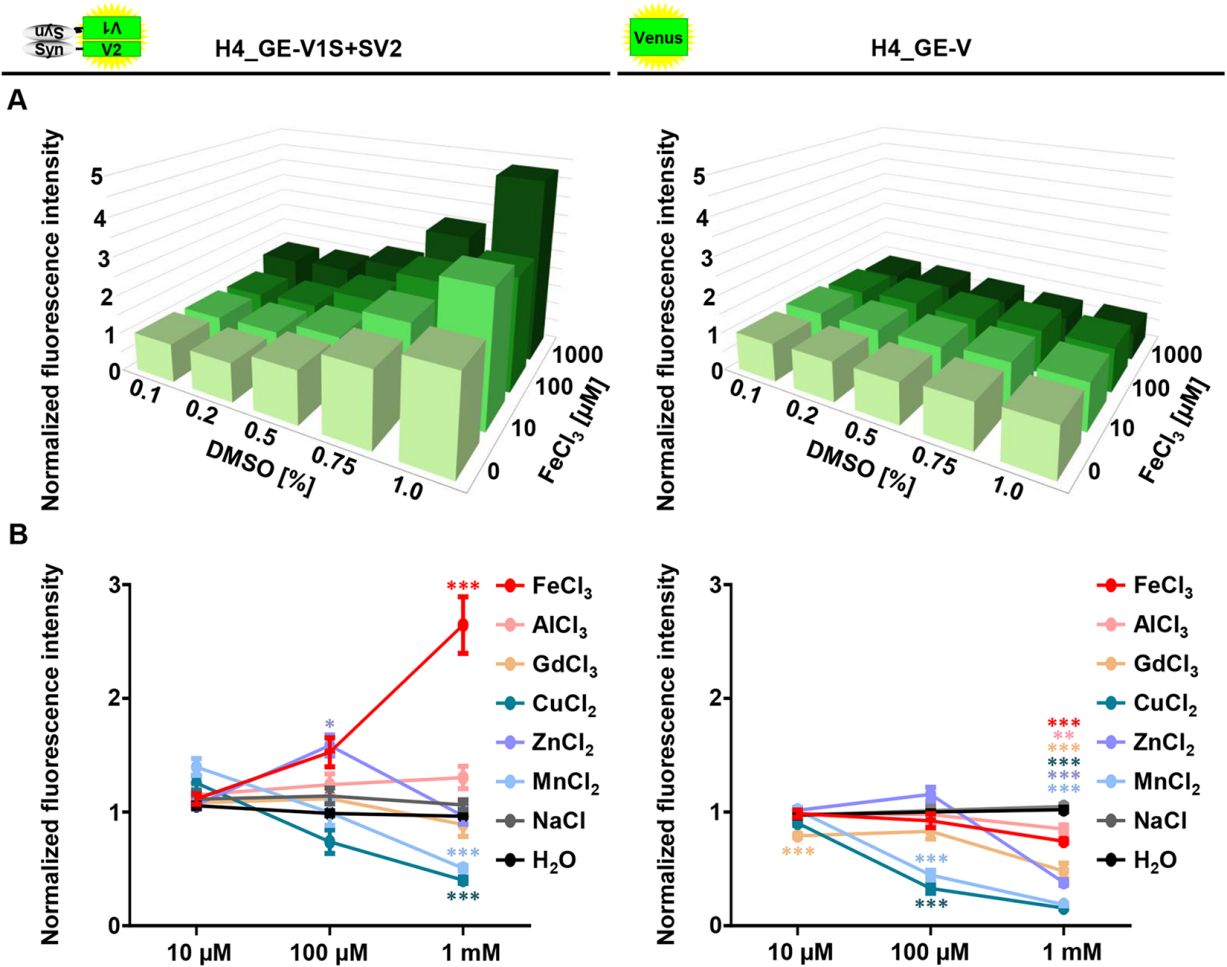
Increased fluorescence intensity in H4_GE-V1S + SV2 cells upon incubation with DMSO and FeCl_3_. H4_GE-V1S + SV2 and H4_GE-V cells were incubated with tebufenozide and different concentrations of DMSO and tri-, di-, or monovalent ions for 3 days. (**A**) Mean cellular Venus fluorescence intensity was increased upon incubation with higher DMSO and FeCl_3_ concentrations in H4_GE-V1S + SV2 cells (left), but not in H4_GE-V cells (right). Bars show mean of three independent experiments. (**B**) Fluorescence intensity increased upon incubation with FeCl_3_ compared to other trivalent (AlCl_3_, GdCl_3_), divalent (CuCl_2_, ZnCl_2_, MnCl_2_), or monovalent (NaCl) ions. All cells were co-incubated with 1% DMSO. Data points show mean of 8 to 9 independent experiments; error bars show SEM. *p’ < 0.05; **p’ < 0.01; ***p’ < 0.001 (Student’s t-test, Bonferroni corrected for multi-testing).

We found an increase in fluorescence intensity with increasing DMSO and FeCl_3_ concentrations in H4_GE-V1S + SV2 cells (Fig. 6, A left) but not in H4_GE-V cells (Fig. 6, A right). The increase in fluorescence intensity was most prominent for incubation with FeCl_3_ compared to other tri-, di-, or monovalent metal ions (Fig. 6B).

**Figure 7:**
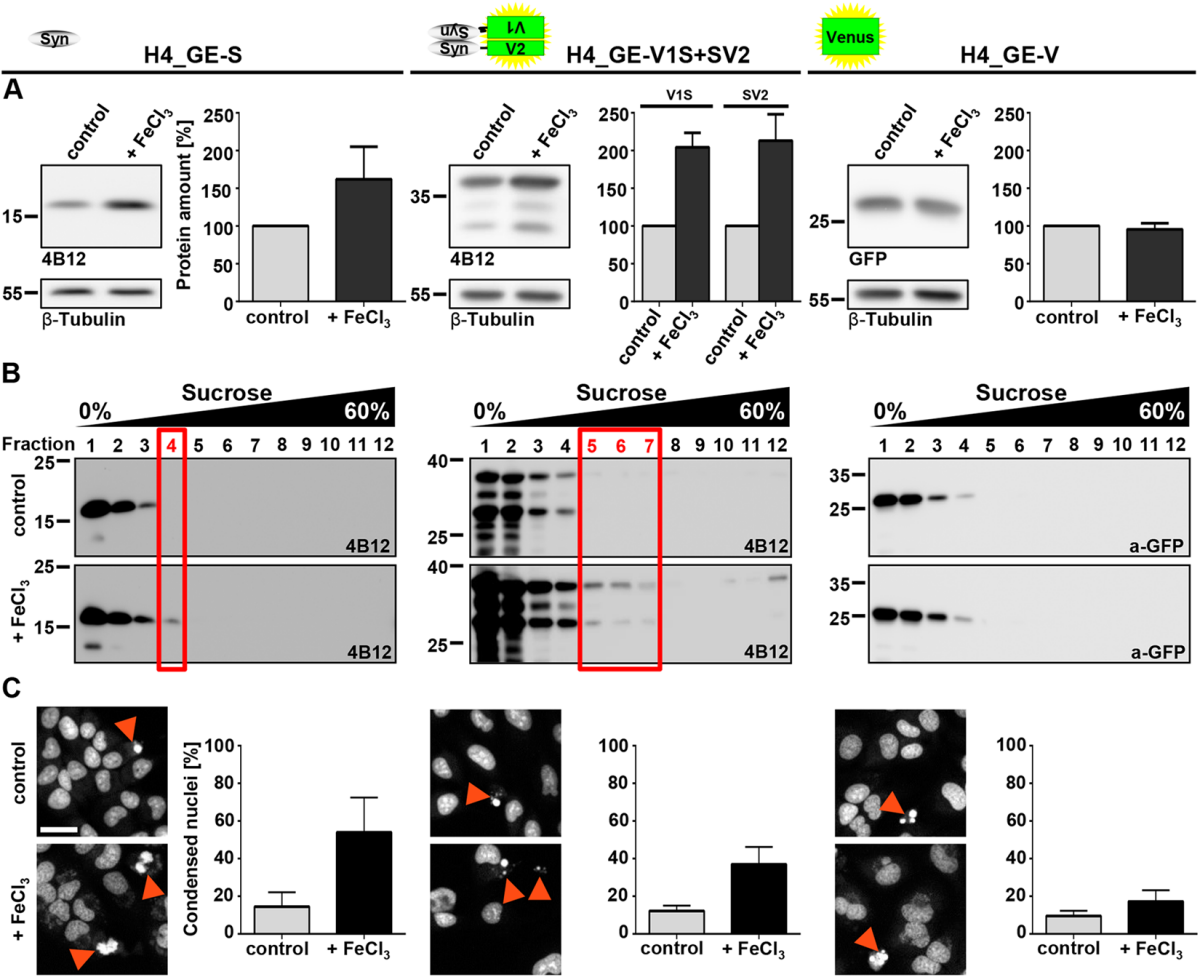
Increased protein amount and aggregation in αSyn-overexpressing H4_GE cells upon incubation with DMSO and FeCl_3_. H4_GE-S, H4_GE-V1S + SV2 and H4_GE-V cells were incubated with 0.75% DMSO and 100 µM FeCl_3_ ( + FeCl_3_) or 0.1% DMSO (control) for 3 days. (**A**) Protein amounts increased in cell lines overexpressing variants of αSyn but not in cells overexpressing Venus upon incubation with 0.75% DMSO and 100 µM FeCl_3_. For quantification signals were normalized to housekeeper. Signals from samples treated with 0.1% DMSO were set to 100%. Bars show mean of 3 to 4 independent experiments and error bars show SEM. Full-length Western blots and additional exposures are shown in Supplementary Figs 9A, 10A, respectively. (**B**) Representative Western blots from cell lysates after sucrose gradient centrifugation of H4_GE-S (left), H4_GE-V1S + SV2 (middle) and H4_GE-V cells (right) incubated with 0.1% DMSO and 0 µM FeCl_3_ (top) or 0.75% DMSO and 100 µM FeCl_3_ (bottom) demonstrating an increase in high molecular weight species in cell lines overexpressing variants of αSyn but not in cells overexpressing Venus following treatment with 0.75% DMSO and 100 µM FeCl_3_ (n = 3). Full-length Western blots and additional exposures are shown in Supplementary Figs 9B, 10B, respectively. (**C**) The fraction of condensed nuclei (red arrowheads) was considerably increased in αSyn-overexpressing cells, but only slightly in Venus-overexpressing cells following incubation with 0.75% DMSO and 100 µM FeCl_3_ suggesting increased cytotoxicity. Scale bar is 50 µm and valid for all panels. Bars show mean of 4 independent experiments, error bars show SEM.

To test if the observed increase in fluorescence intensity in the BiFC assay was due to αSyn aggregation, we also analysed the effect of DMSO and ferric iron on protein amount of cells overexpressing variants of αSyn (H4_GE-S and H4_GE-V1S + SV2) compared to H4_GE-V cells overexpressing Venus. Since we observed many dead and rounded cells upon incubation with 1% DMSO and iron precipitation upon incubation with 1 mM FeCl_3_, we chose the conditions 0.75% DMSO and 100 µM FeCl_3_ versus 0.1% DMSO and 0 µM FeCl_3_. We observed an increase in protein amount in H4_GE-S and H4_GE-V1S + SV2 upon treatment with 0.75% DMSO and 100 µM FeCl_3_ compared to incubation with 0.1% DMSO, while there was no effect on Venus protein amount in H4_GE-V cells (Fig. 7A). An increase in αSyn protein load upon its aggregation has been reported before^26^ and might be due to reduced degradation of aggregated αSyn.

We additionally analysed the aggregation state of αSyn in those cells using sucrose gradient centrifugation. We observed higher molecular αSyn species (occurring in fractions with higher sucrose concentrations) when cells were treated with 0.75% DMSO and 100 µM FeCl_3_ for H4_GE-S and H4_GE-V1S + SV2 cells compared to cells treated with 0.1% DMSO. In contrast, we found no difference between both treatments in H4_GE-V cells (Fig. 7B).

Moreover, we developed an evaluation script for the quantification of cell death relying on the analysis of nuclear morphology. We here observed a strong increase in the fraction of condensed nuclei in H4 cell lines overexpressing αSyn compared to Venus-overexpressing cells upon incubation with 0.75% DMSO and 100 µM FeCl_3_.

In line with the *in vitro* findings from Kostka *et al*.^15^, these findings suggest increased aggregation of αSyn in H4_GE cells upon incubation with high concentrations of DMSO and FeCl_3_ which goes along with increased cytotoxicity. The findings from the BiFC-assay in H4_GE-V1S + SV2 cells were confirmed in cell lines expressing untagged αSyn, further validating the significance of our model system.

## Discussion

We here present the development of H4 cells that stably and inducibly overexpress different constructs, namely wt αSyn (S), the YFP variant Venus (V), αSyn coupled to Venus (SV), and constructs for a BiFC assay (V1S and SV2). Inducible H4_GE and H4_CE^T2^ cells were created using lentiviral transduction resulting in long-term stable gene expression (Supplementary Fig. 1). In order to minimize interexperimental variations of cellular characteristics we applied a strategy where we obtained 300 aliquots of cells with theoretically identical properties for all cell lines. Since a new aliquot is used for every single experiment, a stable transgene expression over a time-course of 23 days (Supplementary Fig. 1) appears to be sufficient.

Since we obtained higher transgene inducibility in pilot experiments for the GE over the CE^T2^ system we performed a detailed characterization of its expression characteristics using the Opera^®^ system and Western blot analyses and obtained almost 100% of transgene expressing cells for all H4_GE cell lines that express fluorescence proteins (H4_mC_GE-S (Fig. 2D), H4_GE-V, H4_GE-SV, and H4_GE-V1S + SV2 (Fig. 3B)). Thus, it is likely that we also obtained a very high fraction of αSyn-expressing cells in the H4_GE-S cell line. We found that mean cellular fluorescence intensity showed a maximum at day 5 or 6 (Fig. 3A). This corresponds to the data obtained from the Western blot evaluation of expression kinetics (Fig. 5). Recently, the development of a comparable model for inducible expression of αSyn in H4 cells has been published^27^. Like for our model, Moussaud *et al*. observed low background expression and an increase in transgene expression over time, reaching a maximum 4 to 6 days after induction (Figs 3, 5). A reliable comparison of the characteristics of both models would require the investigation of both kinds of cell lines in parallel under equal conditions and is difficult from published data alone. However, since the model developed by Moussaud *et al*. relies on transient transfection and a Tet-Off system it is limited to cell lines which are susceptible to transient transfection and which do not apply a tetracyclin based expression system for additional mechanisms. Since our system is applied via lentiviral transduction and is independent of tetracyclin, it holds the additional benefit that it can be applied to cell lines which are not susceptible to transient transfection and use a Tet-On or Tet-Off system for inducible expression of other proteins – including primary cells and LUHMES cells^28,29^.

Interestingly, the fluorescence intensity and the fraction of positive cells increased more slowly in the H4_GE-V1S + SV2 cells compared to H4_GE-V or H4_GE-SV cells (Fig. 3), while protein amounts increased in a comparable fashion (Fig. 5). This might be due to the fact that a detectable fluorescence signal in the BiFC system requires not only expression of a fluorescence protein but also nucleation of αSyn aggregation. This is supported by investigation of the kinetics of protein amount in H4_GE-V1S + SV2 cells which showed a rather exponential increase in fluorescence intensity over time (Fig. 3A) whereas protein amounts of V1S and SV2 increased rather linearly (Fig. 5). Interestingly, Moussaud *et al*. observed αSyn immunoreactivity in H4 BiFC cells which did not display Venus fluorescence suggesting the presence of monomeric αSyn^27^.

Furthermore, we found that fluorescence intensity increased in a tebufenozide-dependent manner upon incubation with 1 nM to 10 µM tebufenozide (Fig. 3A,C). These data are in line with our investigation of protein amounts increasing with tebufenozide concentration using Western blot analysis (Fig. 4) and with previously published data^24^. The decreased fluorescence intensity observed when incubating cells with 100 µM tebufenozide can most likely be explained by precipitation of tebufenozide. Background expression in the absence of tebufenozide was very low or not detectable at all for all H4_GE cell lines (Figs 2A–C, 3A, 4).

BiFC systems have been widely used to study protein-protein interactions in general^21,30^ and oligomerization of αSyn in particular^19,31^, yet protein tags in general possibly interfer with protein functions. It has been shown that Venus protein tags for αSyn complementation assays do neither interfere with the normal cellular distribution of αSyn nor with the polymerization of αSyn, yet, αSyn half-life and oligomer stability appeared to be increased in fluorescence protein-based BiFC cells, probably due to an irreversibility of fragment complementation^27,32–34^. Moreover, protein tags and protein complementation assays have been applied successfully to study αSyn associated aspects including investigation of molecular disease mechanisms^35^, cell based screening assays^27^, and *in vivo*-models^36^. However, to account for a possible effect of the protein tags on the function of αSyn we included extensive control experiments using untagged αSyn. Importantly, cells expressing untagged αSyn showed similar results regarding iron-induced aggregation of αSyn and αSyn-mediated toxicity (Fig. 7) as cells expression the αSyn-Venus fusion constructs. In the fusion system used in this study the Venus fragments are coupled to the N- and C-terminal end of αSyn since this led to highest fluorescence yield^19^, probably emphasizing the detection of oligomers with an anti-parallel assembly of αSyn.

It has been described before that the fluorescence-based αSyn PCA can be used as high-throughput primary assay to screen large libraries^27^ which is in line with our experiment to screen for aggregation enhancers (Fig. 6B). We additionally performed a proof-of-concept experiment using known modulators of αSyn aggregation^5,15,37–41^. As expected, we observed reduced fluorescence intensity using these compounds (Supplementary Fig. 4) further validating the relevance of our model for screening assays. All in all, our findings further support the relevance of BiFC-based inducible αSyn cell models as valuable tools in drug discovery for PD.

Iron has been discussed extensively and controversially as possible risk factor for the development of PD^14^. Taken together, our data suggest an increase in αSyn aggregation in H4_GE cells upon incubation with DMSO and FeCl_3_ (Figs 6, 7) which is well in line with previously published *in vitro* experiments^15^ and thus serves as a proof-of-concept of the presented cell model. Here, the occurrence of high molecular species after sucrose gradient centrifugation was more pronounced in H4_GE-V1S + SV2 cells compared to H4_GE-S cells (Fig. 7B). This may be explained by a stabilizing effect of the complementation of the Venus fragments on oligomeric αSyn species^19^. We also observed an increase in cytotoxicity with αSyn aggregation, which was assessed by label-free quantification of condensed nuclei (Fig. 7C). Interestingly, this was more pronounced in those cells that overexpress untagged αSyn compared to the cells expressing V1S + SV2. This could be due to hampered formation of toxic oligomeric αSyn species as a result of the fluorescence tags or the complementation of the Venus fragments. Taken together, our data on increased aggregation and αSyn-mediated cytotoxicity following exposure to DMSO and FeCl_3_ are well in line with several findings that point towards ferric iron as an important player in the course of pathology further validating the significance of our cell model. Indeed, elevated brain iron levels with a shift in the Fe^2+^/Fe^3+^ ratio in favour of Fe^3+^ have been observed in the SN of PD patients and a neuroprotective effect of iron chelators has been described^42^. Furthermore, iron has been shown to promote aggregation of αSyn resulting in toxicity in cell culture^43^, and incubation with ferric iron has produced pore-forming αSyn oligomers *in vitro*^15,44,45^. While our investigations on iron-dependent αSyn aggregation reproduce earlier *in vitro* findings and validate the value of our cell model, the current study is limited regarding molecular mechanisms by which iron acts on αSyn aggregation. However, it has been described earlier that iron directly interacts with αSyn^44^ and with iron response elements in the 5′ untranslated region in its mRNA^46^.

To date, there is no cure and no disease-modifying therapy for PD available. Therefore, a better understanding of underlying disease mechanisms is inevitable for the development of better diagnostic tools – allowing an earlier and more reliable diagnosis of PD – and for the development of disease-modifying therapies. We here present an important tool for the investigation of αSyn aggregation and the discovery and detailed characterization of novel drug candidates which can finally be translated to clinical application.

## Methods

### Plasmid cloning

Plasmids and primers are summarized in Supplementary Table 1 and Supplementary Table 2, respectively.

Constructs S, V1S, SV2, SV, and V were amplified from plasmids #1-#4 (kind gift from Pamela McLean). S was amplified from plasmid #3 using primers MB8_SV_Hind3_f and MB11_Syn_Not1_r, V1S from plasmid #1 (primers MB24_V1_HindIIIf and MB6_V1S_Not1_r), SV2 from plasmid #2 (primers MB3_SV2_Hind3_f and MB25_V2_NotI_r), SV from plasmid #3 (primers MB8_SV_Hind3_f and MB9_SV_PspOMI_r), V from plasmid #3 (primers MB10_V_Hind3_f and MB9_SV_PspOMI_r).

For lentiviral expression plasmids, constructs were inserted between the HindIII and NotI site of pCR8/GW/TOPO + pCS2-MCS and then transferred to the destination vectors 636, 597, #44, and #92 (Supplementary Table 1), using the Gateway^®^ Technology^47^. Vector #92 was created by amplifying mCherry-NLS from plasmid #87^48^ (pmCherry-NLS was a gift from Martin Offterdinger (Addgene plasmid # 39319)) using primers 42MB_SalI-mCherry_f and 43MB_NsiI_mCherry_r and subcloning between the XhoI and NsiI sites of plasmid #45.

To create plasmid #71, V1S was amplified from plasmid #1 using primers MB24_V1_HindIIIf and MB6_V1S_Not1_r. For plasmid #75, SV2 was amplified from plasmid #2 (primers MB3_SV2_Hind3_f and MB25_V2_NotI_r). Both constructs were subcloned between the HindIII and NotI sites of P12-HA-TGFa-FLAG (Supplementary Table 1)^49^.

### Cell maintenance

HEK293T cells (ATCC, CRL-3216) were maintained in DMEM (PAN, P04–03600) supplemented with 1% glutamine (PAN, P04-80100) and 10% fetal bovine serum (FBS; PAN, P30-3702). H4 human neuroglioma cells (ATCC, HTB-148) were maintained in Opti-MEM (Invitrogen, 31985-070) supplemented with 10% FBS (PAN, P30-3702). Cells were grown at 37 °C in a humidified 95% air/5% CO_2_ atmosphere and passaged twice weekly. All cell lines were regularly tested for mycoplasma contamination.

### Virus production and purification

Packaging plasmids #97 and #98, and corresponding expression plasmids (see Supplementary Table 3) were transfected into HEK293T cells using Lipofectamine^®^ 2000 according to the manufacturer’s instructions.

Virus-containing medium was collected after 48 h and 72 h and centrifuged (3,000 rcf, 15 min). Afterwards, supernatant was filtered using a 0.45-µm filter and centrifuged (22,000 rpm, 2 h, 4 °C, SW28 rotor (Beckman Coulter, 342196), Sorvall Discovery 90SE ultracentrifuge). Pellets were incubated in TBS-5 buffer (50 mM Tris-HCl (pH 7.8), 130 mM NaCl, 10 mM KCl, 5 mM MgCl_2_, 10% BSA (w/V)), sterile filtered) overnight, then centrifuged (800 rcf, 2 min, 4 °C). Supernatant was frozen in 20 µl aliquots at −80 °C.

### Compound testing

100,000 cells/ml were incubated with 0.1% DMSO as a control or 10 µM 3-(1,3-Benzodioxol-5-yl)-5-(3-bromophenyl)-1*H*-pyrazole or anle138c (synthesized as described previously^5^) with a final DMSO concentration of 0.1%. After 24 h, cells were washed with DPBS and fresh normal growth medium was added. Afterwards, cells were transiently transfected with plasmids #71 and #75 using X-tremeGENE^TM^ HP DNA Transfection Reagent according to the manufacturer’s instructions (3 µl/µg_plasmid_; Roche Diagnostics, 06366236001). After 2 h, cells were washed with DPBS and fresh normal growth medium containing the corresponding compound or DMSO was added for 48 h.

### Optimization of the H4 transduction protocol

To optimize the transduction protocol we determined the fraction of fluorescing cells for different protocols manually using the *ImageJ* software.

Protocol A: V99 and V41 were diluted 1:200 in 2 ml of cell-containing medium (50,000 cells/ml).

Protocol B: V99 and V41 were diluted 1:50 in 2 ml cell-containing medium (50,000 cells/ml).

Protocol C: V99 and V41 were diluted 1:12 in 200 µl cell-containing medium (100,000 cells/ml).

Protocol D: V41 was diluted 1:3.5 in 50 µl cell-containing medium. Cells were expanded and maintained as described above. After two weeks, the obtained H4_41 cells were transduced with V99.

For all protocols, cell nuclei were stained with 0.5 µg/ml Hoechst33342 (Invitrogen, H1399) 72 h after transduction.

### Creation of stable inducible H4 cell lines

H4 cells were transduced according to protocol D (described above). For the GE system, cells were transduced with V100, V39 (twice), V41, V42 (twice), V43, V46 (twice) and V120, respectively, resulting in the following cell lines: *H4_100-41 (*H4_GE-SV), *H4_100-4*2_*2*_ (H4_GE-S), *H4_100-43* (H4_GE-V), *H4_100-120 (*H4_mC_GE-S) and *H4_100-39*_2_*-46*_2_ (H4_GE-V1S + SV2). For the CE^T2^ system, cells were transduced with V102, V32, V33, V35, V36, or V37, respectively (see Supplementary Table 3), resulting in the following cell lines: *H4_102-32-33 (*H4_ CE^T2^-V1S + SV2)*, H4_102-35 (*H4_CE^T2^-SV*), H4_102-36 (*H4_ CE^T2^-S*)*, and *H4_102-37 (*H4_ CE^T2^-V*)*.

Cells were expanded and stored in the gas phase of liquid nitrogen. For each experiment a new aliquot was unfrozen to obtain reproducible cell populations.

### Induction of transgene expression

Tebufenozide (Santa Cruz, sc-280110) and (z)-4-OH-tamoxifen (Abcam, ab141943) were diluted in DMSO to a final concentration of 100 mM. To induce transgene expression, cells were incubated with 100 pM to 100 µM and a final DMSO concentration of 0.1%.

### Western blot

Cell pellets were resuspended in lysis buffer (25 mM Tris, 50 mM NaCl, 0.5% Na-deoxycholate, 0.5% Triton X-100, pH = 8.0, protease inhibitor (Roche Applied Science, 04693124001)), incubated at 4 °C and 1,000 rpm on a Thermomixer Comfort (Eppendorf) for 30 min and centrifuged (16,100 rcf, 10 min, 4 °C). Protein concentration was adjusted to 2.08 mg/ml. After blotting, the membranes were incubated overnight with primary antibodies (anti-αSyn (15G7^50^, 4B12 (monoclonal; Hiss, SIG-39730-200)), anti-GFP (Abcam, Ab290), anti-GAPDH (Abcam, ab9485), anti-tubulin (Sigma, T4026), anti-actin (Sigma, A2066)) diluted in 5% milk in TBS-T supplemented with 0.02% NaN_3_. After washing, the membranes were incubated with a secondary antibody (rabbit-anti-rat IgG (H&L) (alkaline phosphatase (AP)-conjugated) (Biomol GmbH, 712-405-002), goat-anti-rabbit IgG (AP-conjugated) (JacksonImmoResearch, 111-055-003), goat-anti-mouse IgG (AP-conjugated) (Cell Signaling Technology, 7056)), goat-anti-rabbit (horseradish peroxidase (HRP)-conjugated) (Cell Signaling Technology, 7074), horse-anti-mouse (HRP-conjugated) (Cell Signaling Technology, 7076)) diluted in 5% milk in TBS-T for 1 h. After washing, the membranes were prepared for signal detection using CDP-Star (Roche Applied Science, 12041677001) for AP coupled antibodies or using the Clarity^TM^ Western ECL Substrate (BioRad, 1705060) for HRP coupled antibodies, respectively. Chemiluminescence signals were detected with the ChemiLux camera system and the ChemoStar software (Intas).

### Sucrose gradient centrifugation

Cell pellets were resuspended in 40 µl SC lysis buffer (50 mM Tris (pH 7.4), 175 mM NaCl, 0.1% NP-40, 1x protease inhibitor) and incubated on ice for 15 min twice before centrifugation (17,000 rcf, 1 min, 4 °C). Concentration of the supernatant adjusted to 50–200 µg of total protein in 200 µl in 50 mM Tris (pH 7.4), 175 mM NaCl, and 0.1% NP-40. Centrifuge tubes were filled with buffer A (50 mM Tris-HCl (pH 7.5), 0.1% NP-40, 0–60% Sucrose) in layers in the given order: 200 µl 60% sucrose, 400 µl each of 50–10% sucrose, 200 µl sample without sucrose. The samples were ultracentrifuged in a Sorvall WX Ultra 90 (Thermo Scientific) (40,000 rpm, SW60 rotor (Beckmann Coulter), 70 min, 4 °C) and 12 fractions of 200 µl each were collected, mixed with 800 µl of 12.5% trichloroacetic acid (TCA) and incubated at 20 °C overnight. After centrifugation at 4 °C and 20,000 rcf for 15 min, pellets were washed with 1 ml precooled acetone (−20 °C) and centrifuged at 4 °C and 20,000 rcf for 15 min. Pellet was allowed to dry at RT for 5 min. 30 µl of 5x Lämmli sample buffer were added and samples were shaken at 30 °C and 1,400 rpm for 10 min followed by incubated at 96 °C for 5 min.

### Confocal single particle spectroscopy

Cell lysis and single particle spectroscopy was performed as described previously^51^ using an Insight^TM^ Reader (Evotec-Technologies). The total brightness (Itot) was adapted to 50 to 100 kHz by dilution in RIPA buffer (50 mM Tris-HCl, pH 7.6, 1% NP-40, 150 mM NaCl, 1 mM EDTA). Laser power of the argon-ion laser (488 nm) was adjusted to 200 µW. Measurement was performed for 10–15 seconds at RT, samples were scanned in a length of 100 µm with a frequency of 50 Hz of the beam scanner and a displacement of 2,000 µm of the sample carrier. Data were analysed by fluorescence intensity distribution analysis^52^ (FIDA) using the FCSPP evaluation software version 2.0 (Evotec-Technologies).

### High content imaging (opera^®^)

High content screening was performed using the Opera^®^ high-throughput confocal imaging platform (PerkinElmer Cellular Technologies GmbH, Hamburg, Germany). Nuclei were stained by with 5 µM Draq5 (Thermo Fisher Scientific, 62252). All measurements were performed at RT using the 20x air objective. A detailed description of the setup and data analysis can be found in the supplementary Methods (Supplementary Fig. 11).

Fluorescence of Venus in the cytoplasm was excited using the 488-nm laser with a laser power of 7,110 µW and focus height was set to −8.0 or −9.0 µm relative to the autofocus level; all values of focus height were chosen in order to obtain optimal fluorescence yield. Fluorescence was detected using Camera 1 with an exposure time of 200 ms or 320 ms and twofold binning using a 520/35 filter. Fluorescence of mCherry was excited using the 561-nm laser with a laser power of 3,240 µW and focus height was set to −13.0 µm relative to the autofocus level; fluorescence was detected using Camera 2 with an exposure time of 7,000 ms and twofold binning using a 600/40 filter. Fluorescence of Draq5 in the nucleus was excited using the 640-nm laser with a laser power of 3,830 µW and focus height was set to −12.0 or −13.0 µm relative to the autofocus level; fluorescence was detected using Camera 3 with an exposure time of 40 to 400 ms and twofold binning using a 690/50 filter.

For data analysis, an automated image analysis tool was developed using the Acapella^®^ software (PerkinElmer Cellular Technologies GmbH) in order to quantify cellular Venus fluorescence and the fraction of condensed nuclei (Supplementary Fig. 12).

The datasets generated during the current study are available from the corresponding author on reasonable request.

## Supplementary information

Supplementary Information
