# Supplementary material for: Iron-mediated aggregation and toxicity in a novel neuronal cell culture model with inducible alpha-synuclein expression
Source: Sci Rep. 2019 Jun 24;9:9100. doi: 10.1038/s41598-019-45298-6 (PMC6591385; doi:10.1038/s41598-019-45298-6)
Supplement: Supplementary file 1 — Supplementary Information [file 41598_2019_45298_MOESM1_ESM.pdf]

Iron-mediated aggregation and toxicity in a novel neuronal cell culture model with inducible alpha-synuclein expression

Martin Bartels[1,2], Daniel Weckbecker[3], Peer-Hendrik Kuhn[4], Sergey Ryazanov[5,6], Andrei Leonov[3,5,6], Christian Griesinger[5,6], Stefan F. Lichtenthaler[7,8], Kai Bötzel[2] and Armin Giese[1*]

From the [1]Center for Neuropathology and Prion Research, Ludwig-Maximilians-University, Munich, Germany; [2]Department of Neurology, Klinikum der Universität München, Munich, Germany; [3]MODAG GmbH, Wendelsheim, Germany; [4]Institute of Pathology, TUM School of Medicine, Technical University of Munich, Munich, Germany; [5]Center for Nanoscale Microscopy and Molecular Physiology of the Brain, Georg-August-University Göttingen, 37073 Göttingen, Germany; [6]Department of NMR-based Structural Biology, Max Planck Institute for Biophysical Chemistry, 37077 Göttingen, Germany; [7]German Center for Neurodegenerative Diseases (DZNE), and Munich Cluster for Systems Neurology (SyNergy), Munich, Germany; [8]Neuroproteomics, School of Medicine, Klinikum rechts der Isar, and Institute for Advanced Science, Technical University of Munich, 81675 Munich, Germany

[*]To whom correspondence should be addressed: Armin Giese, Center for Neuropathology and Prion Research, Ludwig-Maximilians-University, Munich, Germany, armin.giese@med.uni-muenchen.de; Tel. 0049 89 2180 78007; Fax. 0049 89 2180 78037

Supplements

Supplementary Tables

Supplementary Table 1: List of plasmids

Information on plasmids used in this study.

ID	Insert	Backbone
#1	V1S	pCS2
#2	SV2	pCS2
#3	SV	pCS2
#4	Venus	pCS2
#32	SV2	F2P-Delta Zeo-LoxP-Ko.Puro-LoxP-STNST-GW(DEST) (636)
#33	V1S	F2P-Delta Zeo-LoxP-Ko.Puro-LoxP-STNST-GW(DEST) (636)
#35	SV	F2P-Delta Zeo-LoxP-Ko.Puro-LoxP-STNST-GW(DEST) (636)
#36	αSyn	F2P-Delta Zeo-LoxP-Ko.Puro-LoxP-STNST-GW(DEST) (636)
#37	Venus	F2P-Delta Zeo-LoxP-Ko.Puro-LoxP-STNST-GW(DEST) (636)
#39	V1S	F2-Delta Zeo-Kozak-Puro-5XUAS-E1b-(GW)Dest (597)
#41	SV	F2-Delta Zeo-Kozak-Puro-5XUAS-E1b-(GW)Dest (597)
#42	αSyn	F2-Delta Zeo-Kozak-Puro-5XUAS-E1b-(GW)Dest (597)
#43	Venus	F2-Delta Zeo-Kozak-Puro-5XUAS-E1b-(GW)Dest (597)
#44	ccdB	F2-Delta Zeo-Kozak-Hygro-5XUAS-E1b-(GW)Dest
#45	ccdB	F2-Delta Zeo-Kozak-Zeo-5XUAS-E1b-(GW)Dest
#46	SV2 in 44	F2-Delta Zeo-Kozak-Hygro-5XUAS-E1b-(GW)Dest
#71	V1S	P12-HA-TGFa-FLAG
#75	SV2	P12-HA-TGFa-FLAG
#87	mCherry-NLS	pmCherry-C1
#92	ccdB	F-Delta Zeo-Kozak-mCherry-NLS-5X UAS-E1b-(GW)Dest
#97	psPAX2	
#98	VSV-G	pcDNA3.1
#99	Zeo-GAL4-VP16	FUGW
#100	Zeo-GAL4-VP16-EcR	FUGW
#102	F2U-Delta Zeo-iCre_ER^{T2}	FUGW
#120	S	F-Delta Zeo-Kozak-mCherry-NLS-5X UAS-E1b-(GW)Dest
597	ccdB	F2-Delta Zeo-Kozak-Puro-5XUAS-E1b-(GW)Dest
636	ccdB	F2P-Delta Zeo-LoxP-Ko.Puro-LoxP-STNST-GW(DEST) (636)
---	Empty	pCR8/GW/TOPO+pCS2-MCS

Supplementary Table 2: List of primers

Information on primers used for molecular cloning in this study.

Primer Name	Sequence (5’-3’)
MB3_SV2_Hind3_f	GATCAAGCTTGCCACCATGGATGTATTCATGAAAGG
MB6_V1S_Not1_r	GATCGCGGCCGCTTAGGCTTCAGGTTCGTAG
MB8_SV_Hind3_f	GCGCAAGCTTGCCACCATGGATGTATTCATGAAAGG
MB9_SV_PspOMI_r	GATCGGGCCCTCTACAAATGTGGTATGGCTG
MB10_V_Hind3_f	GATAAAGCTTGCCACCATGGTGAGCAAGGGCGAGG
MB11_Syn_Not1_r	GATCGCGGCCGCCTAGGCTTCAGGTTCGTAGTCTTG
MB24_V1_HindIIIf	GATCAAGCTTGCCACCATGGTGAGCAAGGGCGAGGAGC
MB25_V2_NotI_r	GATCGCGGCCGCTTACTTGTACAGCTCG
42MB_SalI_mCherry_f	GATCGTCGACGCCACCATGGTGAGC
43MB_NsiI_mCherry_r	GATCATGCATTTATCTAGATCCGGTGGATCC

Supplementary Table 3: List of viruses

Plasmids used for production of infectious but replication-incompetent viruses. Co-transfection of HEK293T cells with viruses #97 and #98 and an expression plasmid resulted in production of viruses shown in the right column.

Plasmid ID	Kind of Plasmid	Insert	Comment	Virus ID
#97	Packaging	psPAX2	Encodes for viral enzymes gag-pol to generate lentiviruses	
#98	Packaging	VSV-G	Encodes for the envelope glycoprotein VSV-G from Vesicular Stomatitis Virus (broad tropism)	
#99	Expression	Zeo-GAL4-VP16	Constitutive GAL4 driver	V99
#100	Expression	Zeo-GAL4-VP16-EcR	Inducible GAL4_EcR driver (+tebufenozide)	V100
#102	Expression	F2U-Delta Zeo-iCre_ER^{T2}	Inducible Cre_ER^{T2} driver (+4-OH-tamoxifen)	V102
#32	Expression	SV2	Cre-loxP receiver (puromycin resistance)	V32
#33	Expression	V1S	Cre-loxP receiver (puromycin resistance)	V33
#34	Expression	V1	Cre-loxP receiver (puromycin resistance)	V34
#35	Expression	SV	Cre-loxP receiver (puromycin resistance)	V35
#36	Expression	S	Cre-loxP receiver (puromycin resistance)	V36
#37	Expression	V	Cre-loxP receiver (puromycin resistance)	V37
#39	Expression	V1S	GAL4-UAS receiver (puromycin resistance)	V39
#41	Expression	SV	GAL4-UAS receiver (puromycin resistance)	V41
#42	Expression	S	GAL4-UAS receiver (puromycin resistance)	V42
#43	Expression	V	GAL4-UAS receiver (puromycin resistance)	V43
#46	Expression	SV2	GAL4-UAS receiver (hygromycin resistance)	V46
#120	Expression	S	GAL4-UAS receiver (mCherry-NLS)	V120

Supplementary Figures

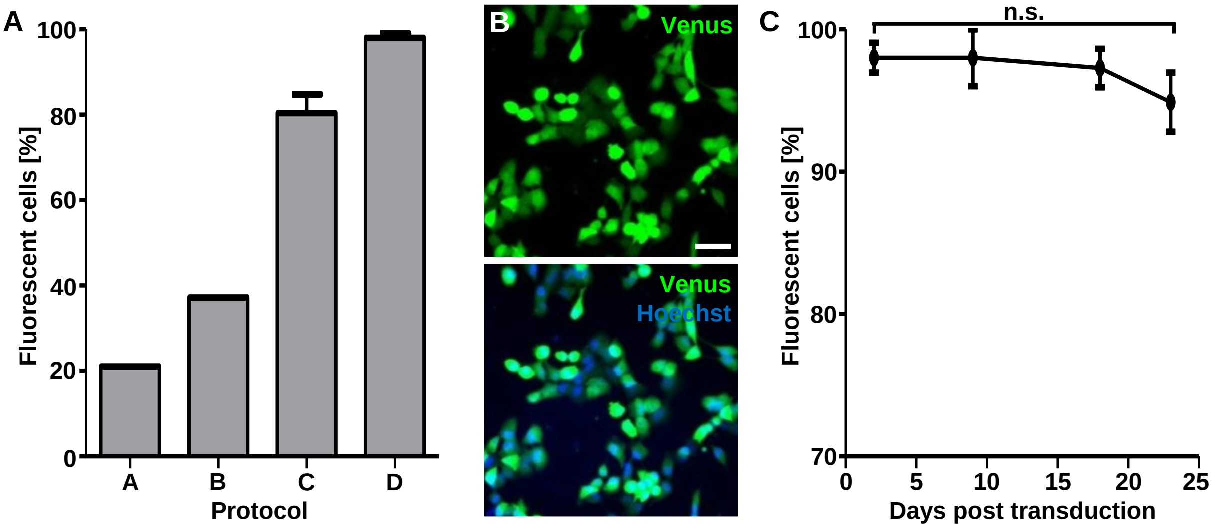

Supplementary Figure 1. Stable and highly efficient transduction of H4 cells following protocol D. H4 cells were transduced with the constitutive GAL4 driver virus (V99) and the UAS-SV receiver virus (V41) according to protocols A-D. (A) Quantification of the fraction of Venus-positive cells. (B) Representative images obtained for protocol D. Nuclei were stained with Hoechst33342. Scale bar is 100 µm and valid for both panels. (C) The fraction of positive cells was not significantly changed for 23 days after transduction (Fisher's exact test, n.s.: $p>0.05$). Data points represent mean of 3 to 4 wells; error bars show SEM; at least 300 cells were counted.

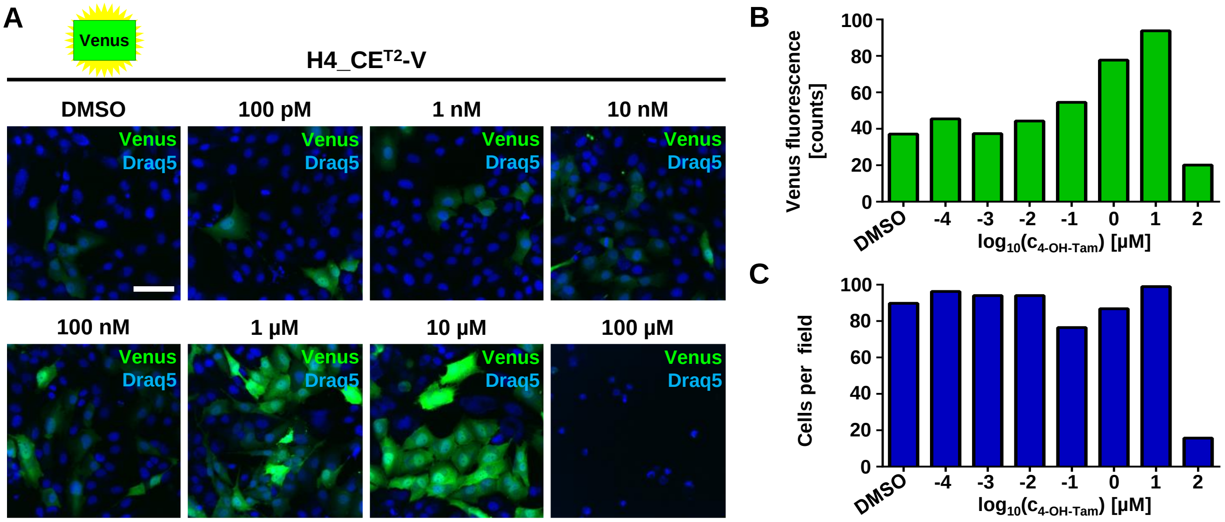

Supplementary Figure 2. Maximum transgene induction in H4_CE^{T2}-V cells was achieved by 10 µM 4-OH-tamoxifen. H4_CE^{T2}-V cells were incubated with different concentrations of 4-OH-tamoxifen ranging from 100 pM to 100 µM or DMSO as control for 72 h to determine its optimal concentration. (A) Fluorescence images showed increasing fluorescence with increasing concentration of 4-OH-tamoxifen up to a concentration of 10 µM. Nuclei were stained with Draq5. Scale bar is 100 µm and valid for all panels. (B) Quantification of fluorescence intensity using the Opera® system. A maximum in fluorescence intensity was observed upon incubation with 10 µM 4-OH-tamoxifen. Bars in B and C show mean of four wells. (C) Quantification of cell number using the Opera® system. Incubation with 100 µM 4-OH-tamoxifen led to decreased cell number whereas all other concentrations showed no adverse effect on cell number.

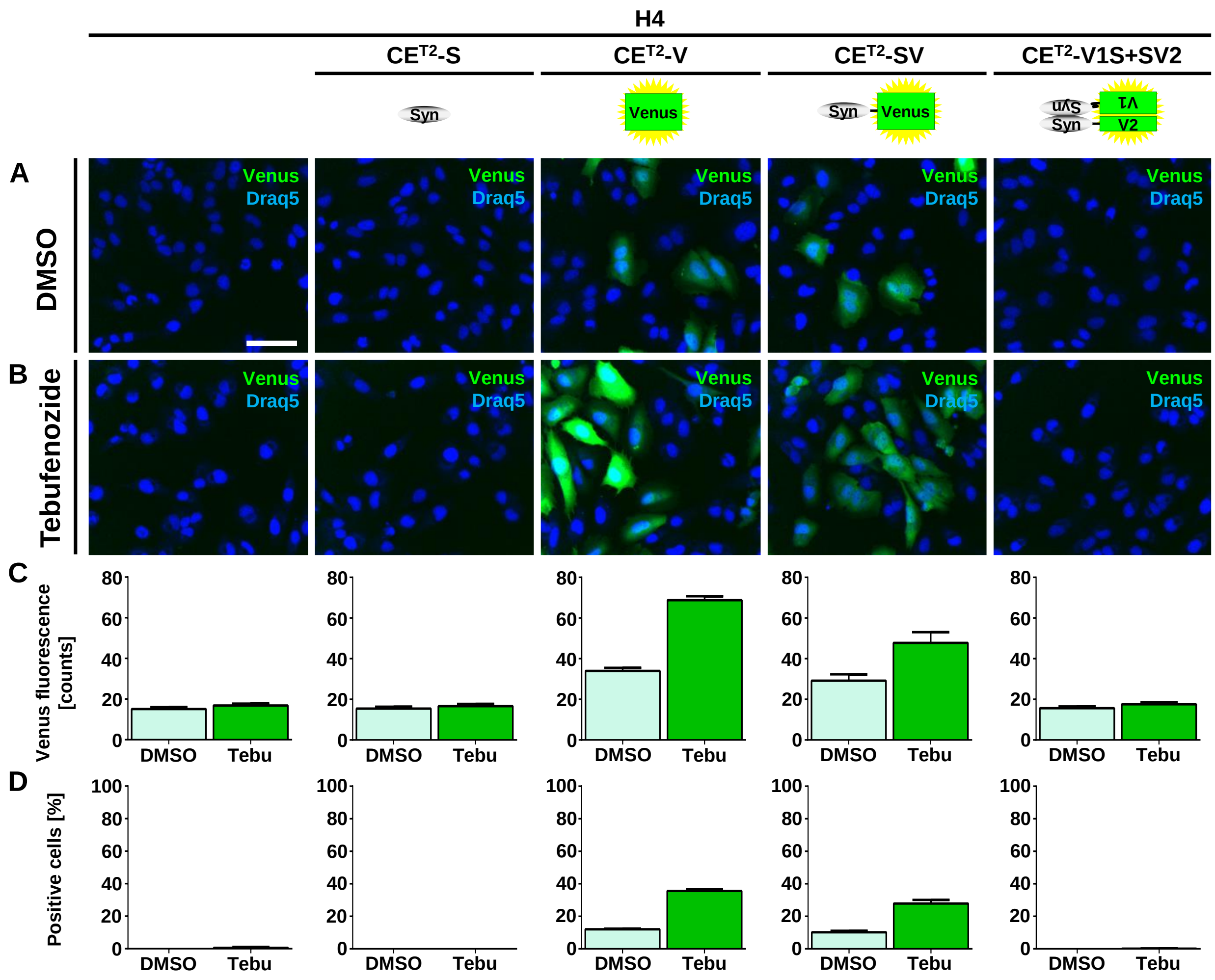

Supplementary Figure 3. Induction of transgene expression in H4_CE^{T2} cells. The generated H4_CE^{T2} cell lines were incubated with 0.1% DMSO or 10 µM 4-OH-tamoxifen for 48 h. (A) Representative fluorescence images of cells incubated with 0.1% DMSO. Nuclei were stained with Draq5. Scale bar is 100 µm and valid for all panels in A and B. (B) Representative fluorescence images of cells incubated with 10 µM 4-OH-tamoxifen. (C) Quantification of the mean cellular Venus fluorescence intensity of H4 cells upon incubation with DMSO or 4-OH-tamoxifen using the Acapella® software. Bars in C ans D show mean of 3 independent experiments; error bars show SEM; Tam: 4-OH-tamoxifen. (D) Quantification of the fraction of Venus-positive H4 cells upon incubation with DMSO or 4-OH-tamoxifen using the Acapella® software. Quantification was performed by setting an arbitrary threshold for Venus fluorescence intensity.

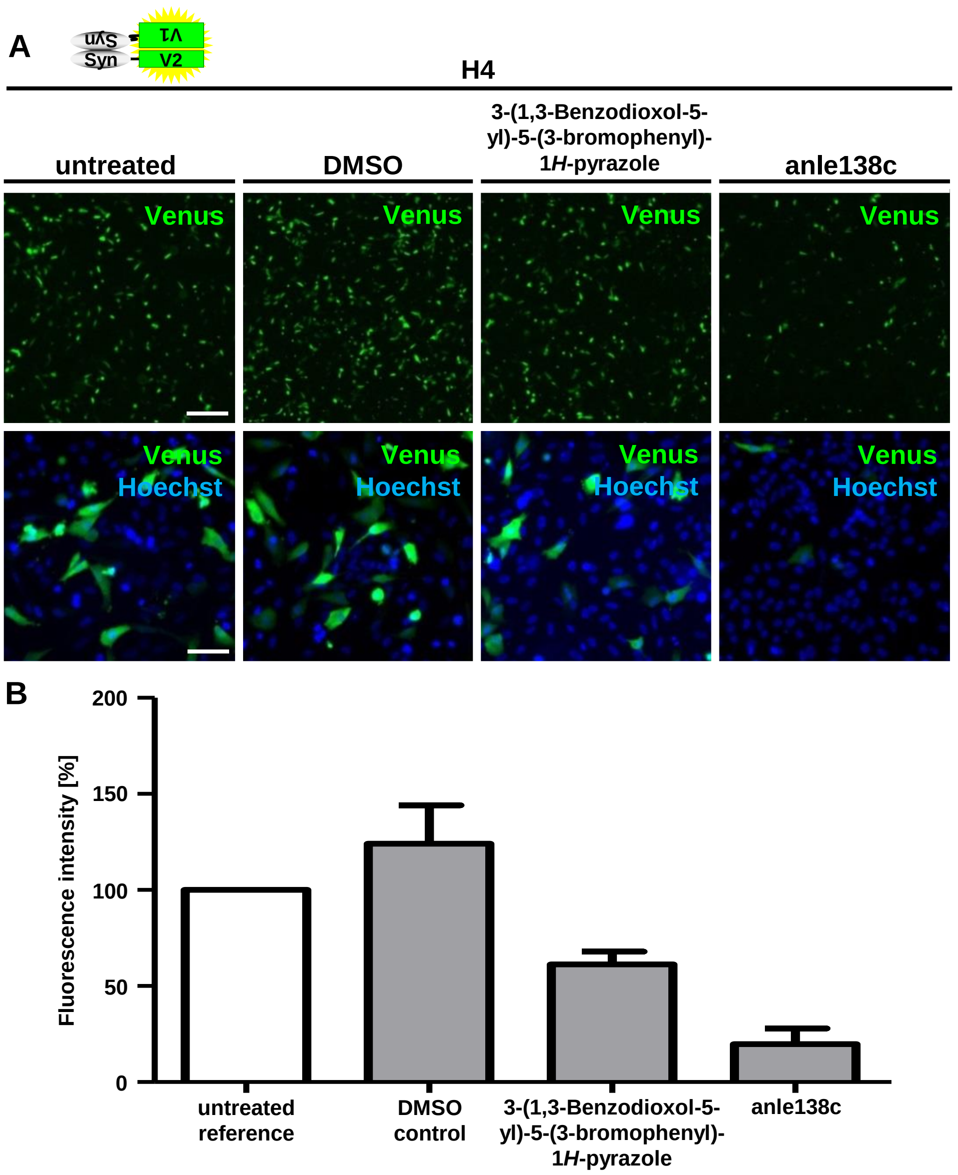

Supplementary Figure 4. Reduced fluorescence intensity in the BiFC system as result of aggregation inhibitors. H4 cells were transiently transfected with the BiFC plasmids and left untreated or were incubated with DMSO (control), 3-(1,3-Benzodioxol-5-yl)-5-(3-bromophenyl)-1*H*-pyrazole, anle138c, or baicalein. (A) Fluorescence images showed a decrease in fluorescence intensity upon treatment with 3-(1,3-Benzodioxol-5-yl)-5-(3-bromophenyl)-1*H*-pyrazole, anle138c, and baicalein compared to treatment with DMSO. Nuclei were stained with Hoechst33342. Scale bar in top left panel is 500 µm and valid for images in the first row. Scale bar in the left panel of the second row is 100 µm and valid for images in the second and third row. (B) Cell lysates were analyzed using single particle spectroscopy. Total fluorescence intensity was increased in DMSO-treated cells compared to untreated cells. Fluorescence intensity was decreased upon treatment with 3-(1,3-Benzodioxol-5-yl)-5-(3-bromophenyl)-1*H*-pyrazole or anle138c. Bars show mean of 2 individual experiments, error bars show SEM.

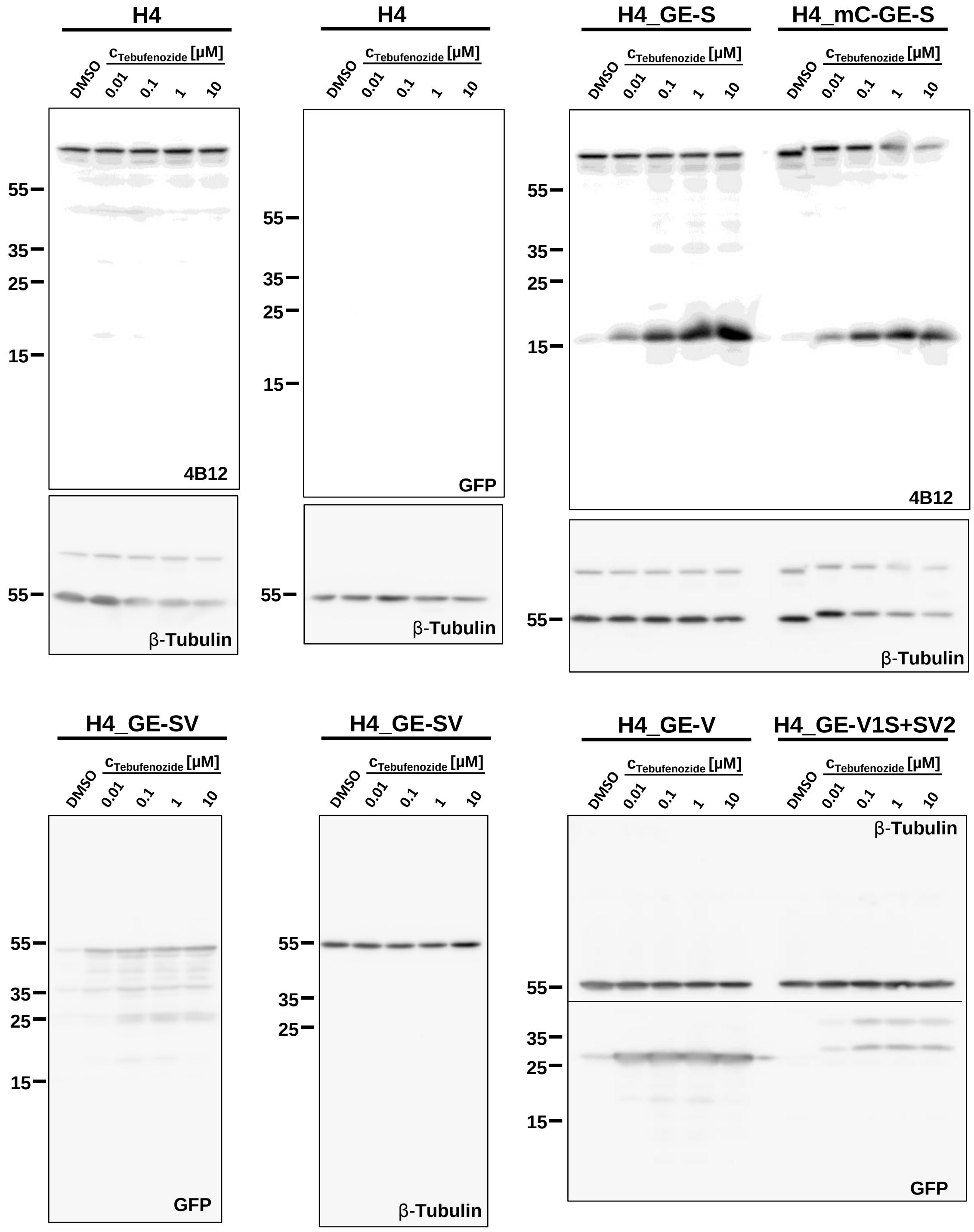

Supplementary Figure 5. Full length Western blots corresponding to the panels shown in Figure 4. For details see legend of Figure 4. Blots from H4, H4_GE-S, and H4_mC-GE-S were stripped after incubated with 4B12 or GFP antibody and afterwards incubated with β-Tubulin antibody. For H4_GE-SV, housekeeper was detected on a separate blot. For H4_GE-V and H4_GE-V1S+SV2 the blot was cut at 40 kDa and incubated with β-Tubulin or GFP antibody, respectively.

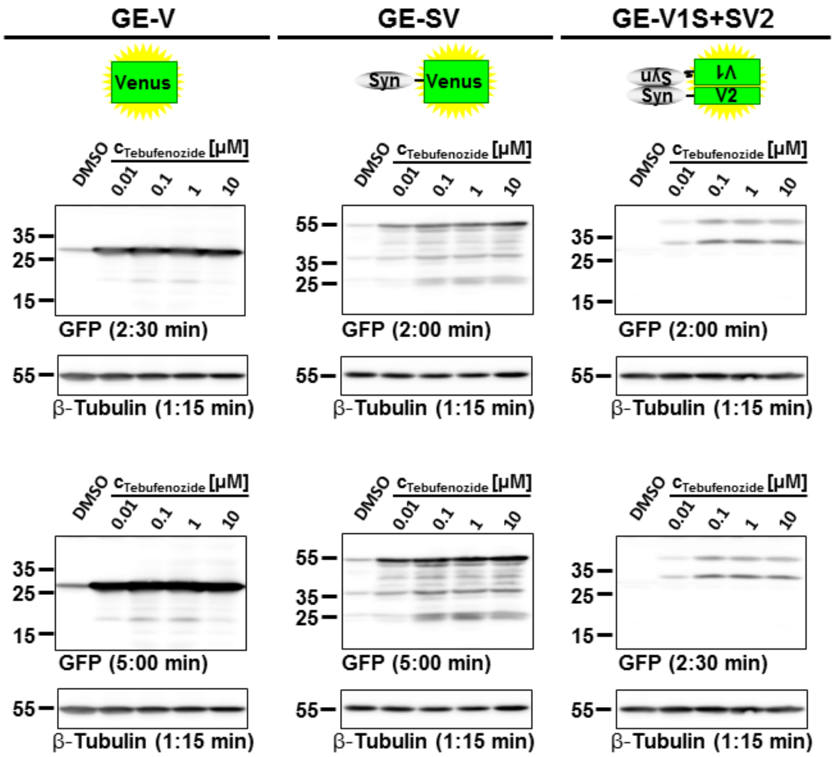

Supplementary Figure 6. Additional exposures of Western blots shown in Figure 4. For details see legend of Figure 4. For H4_GE-SV, housekeeper β-Tubulin was detected on a separate blot. For H4_GE-V and H4_GE-V1S+SV2 the blot was cut at 40 kDa and incubated with β-Tubulin or GFP antibody, respectively.

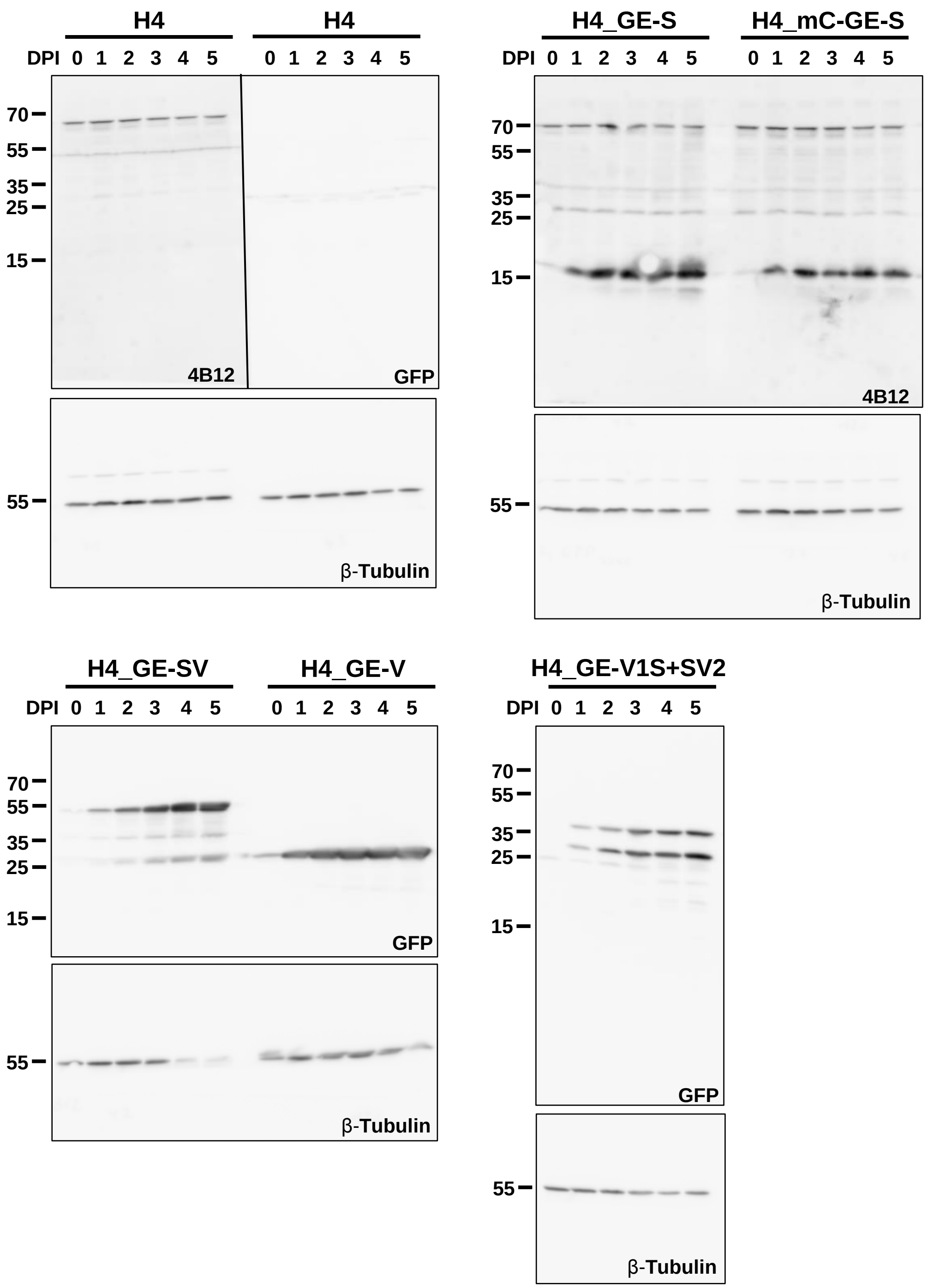

Supplementary Figure 7. Full length Western blots corresponding to the panels shown in Figure 5. For details see legend of Figure 5. All blots were stripped after incubated with 4B12 or GFP antibody and afterwards incubated with β-Tubulin antibody.

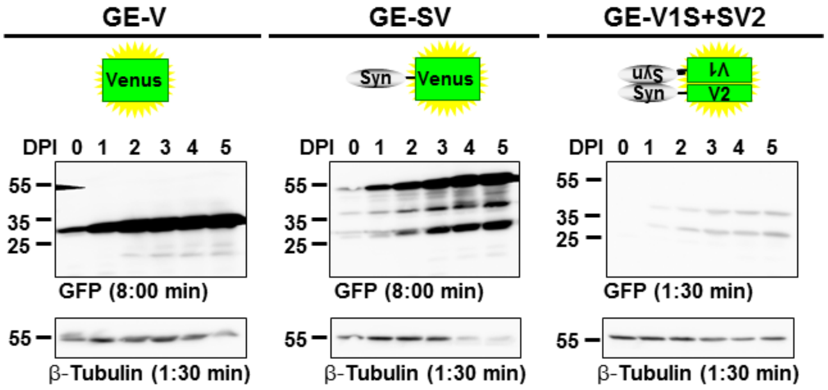

Supplementary Figure 8. Additional exposures of Western blots shown in Figure 5. For details see legend of Figure 5. All blots were stripped after incubation with GFP antibody and afterwards incubated with β-Tubulin antibody.

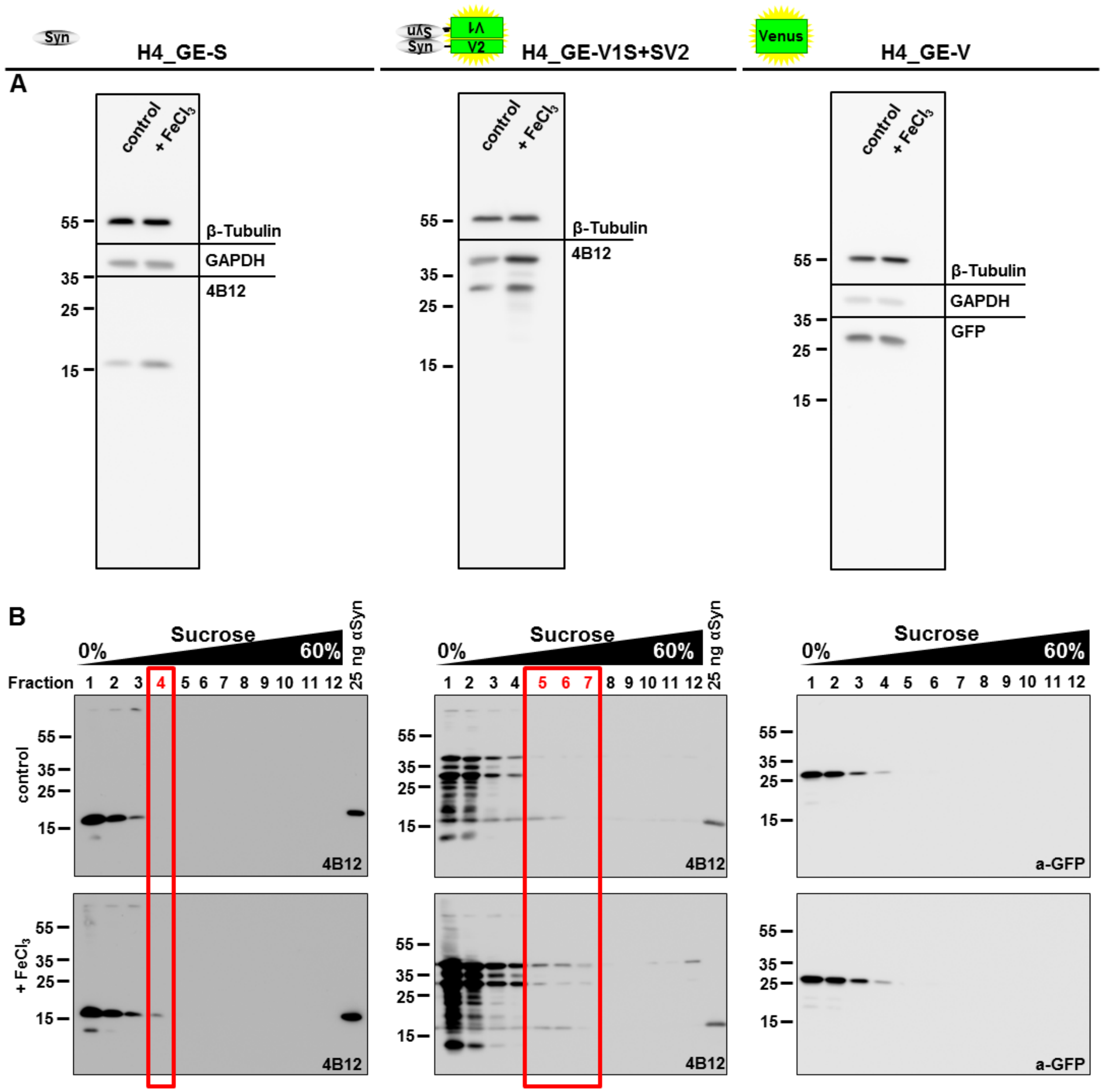

Supplementary Figure 9. (A) Full length Western blots corresponding to the panels shown in Figure 7 A. All blots were cut at 35 kDa and/or at 40 kDa and incubated with β-Tubulin, GAPDH, 4B12 or GFP antibody, respectively. (B) Full length Western blots corresponding to the panels shown in Figure 7 B. 25 ng of alpha-synuclein (αSyn) were loaded as control. For details see legend of Figure 7.

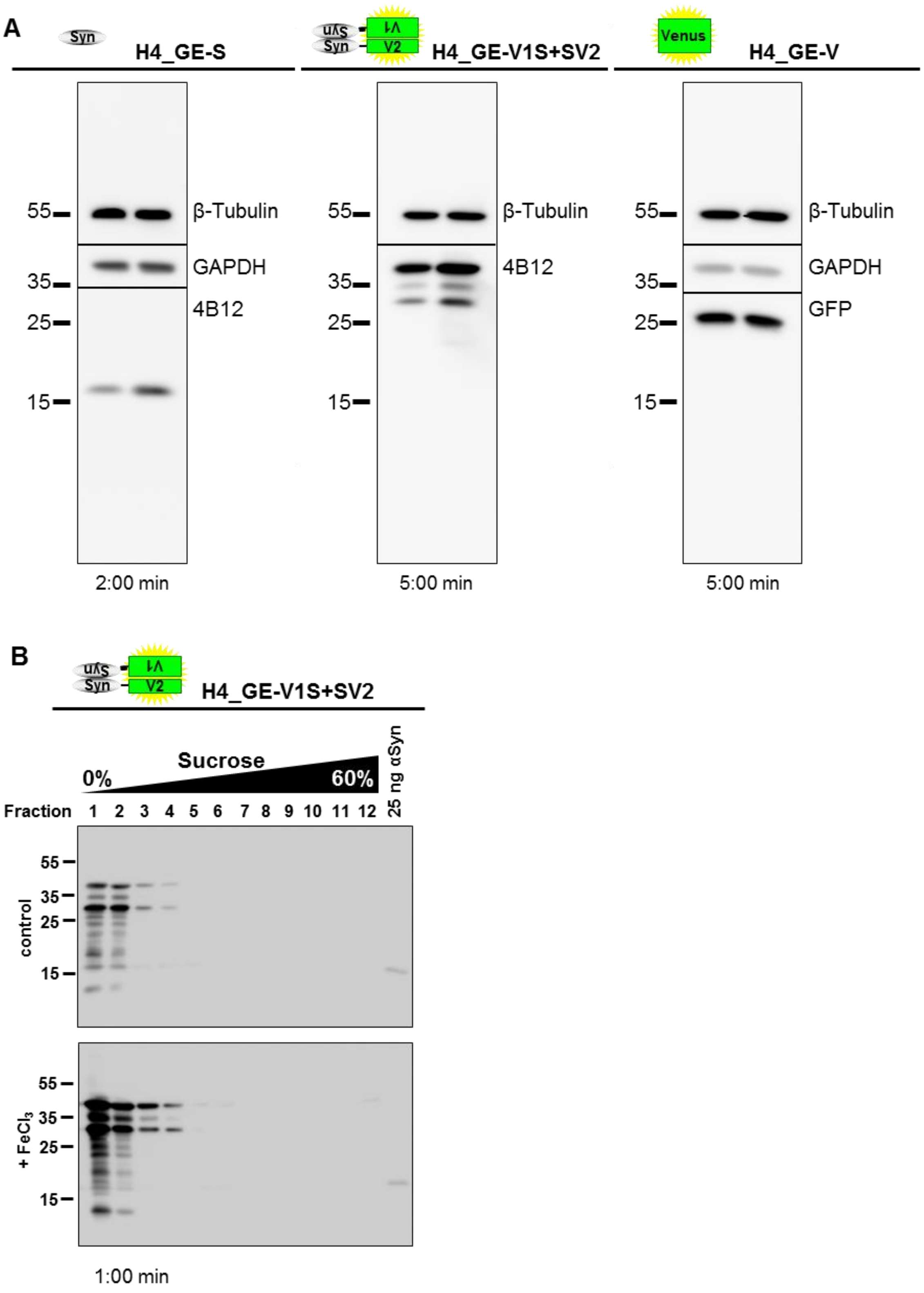

Supplementary Figure 10. Additional exposures of Western blots shown in Figure 7. All blots in (A) were cut at 35 kDa and/or at 40 kDa and incubated with β-Tubulin, GAPDH, 4B12 or GFP antibody, respectively. In (B), 25 ng of alpha-synuclein (αSyn) were loaded as control. For details see legend of Figure 7.

Supplementary Methods

Measurement Setup of the Opera® System

Prior to measurement, nuclei were stained by incubating the cells for 15 min at 37°C in a humidified 95% air/5% CO_2 atmosphere with Draq5 (Thermo Fisher Scientific, 62252) diluted 1:1,000 in medium to a final concentration of 5 µM. High content screening was performed using the Opera® high-throughput confocal imaging platform (PerkinElmer Cellular Technologies GmbH, Hamburg, Germany). In this setup (Supplementary Figure 8 A), laser light can be emitted at four different wavelengths: 442 nm, 488 nm, 561 nm, and 640 nm. The light passes the Nipkow unit with the primary dichroic mirror depending on the filters and can be focused on the sample carrier using four different objectives: one 20-fold air objective (20x_Air_LUCPLFLN_NA=0.45), and three different water immersion objectives: 20xW_UAPO20xW3/340_NA=0.7, 40xW_PlanAPO_40xWLSM_NA=0.9, and 60xW_UPLAPO_60x_ NA=1.2. Fluorescent dyes or proteins in the sample carrier will emit photons upon excitation by laser light with corresponding wavelengths. Since this fluorescence light is emitted in all directions, a part of it passes the objective and follows the way of the exciting laser light retrogradely until it is reflected by the primary dichroic mirror. Depending on its wavelength, fluorescence can finally be detected by three different cameras. Camera 3 can detect fluorescence excited with 640 nm. Fluorescence excited with all other lasers is mirrored at the detection dichroic mirror 2 (“Detection dichro 2”) and subdivided according to its wavelength at the detection dichroic mirror (“Detection dichro”). Depending on the filter settings applied, light of given wavelengths is finally detected using Camera 1 or Camera 2.

In order to reduce background signal, the light in a conventional confocal fluorescence microscope would usually pass a pinhole which is inserted at a position corresponding to the intermediate image of a conventional optical microscope and would thus exclude signal from fluorescent particles below or above the focus plane. In the Opera® high-throughput confocal imaging platform the confocal pinholes are replaced by a Nipkow unit (Supplementary Figure 8 B). In this setup, the exciting laser light is focused by a microlense array before it passes the primary dichroic mirror, the pinhole disk, and the objective to finally excite fluorescent dyes or proteins in the sample. The fraction of fluorescence light that is guided retrogradely passes the pinhole disk, a spinning disk with holes arranged in an arched profile. The microlenses on the microlense array and the pinholes on the pinhole disk are arranged in a similar manner. Due to the rotation of the microlense array and the pinhole disk (with a speed of 25 revolutions per second) not a single point is detected but an arched area of the image. Due to the arrangement of the microlenses and the pinholes and the spinning of those disks, the single arched areas will add up to a complete image of the focal area.

The autofocus function enables a fully automated acquisition of fluorescence images. Here, characteristic reflections of the autofocus laser are detected when passing the bottom of the multiwell plate. Afterwards, laser light is focused on a defined height above the bottom of the multiwell plate depending on the desired image plane.

Adjustment of the Opera® System

Prior to each experiment a reference image and a skew analysis have to be applied in order to compensate for systemic errors of the optics and the CCD cameras.

Uneven brightness distribution within an image was compensated for by using the “reference image” function. Using the Opera® adjustment plate, an image of free floating fluorescence dye was acquired. This should theoretically lead to evenly distributed fluorescence intensity within the image field. Practically, we observed higher intensity in the center of the image and decreasing intensity towards the

edges. This effect is counteracted by the flat-field correction algorithm which (simplified) works by multiplying the recorded image with the inverted reference image.

The mechanical alignment of the CCD cameras alone is subject to variations (for example due to changes in temperature) and not sufficient to provide an adequate overlay of high resolution images obtained from more than one camera. To counteract this phenomenon the "skew analysis" function was applied. The Opera® adjustment plate provides wells with beads with different diameters for the different objectives: 10 µm for 10-fold, 5 µm for 20-fold, and 2.5 µm for 40-fold and 60-fold. Images of these beads are recorded for all excitation wavelengths and all detection cameras. Afterwards, a theoretical optimal overlay of the obtained images is calculated. This optimization is later on applied to all images of one measurement.

In order to set up an automated experiment, the kind of multiwell plate and the required objective were defined. Wavelengths and intensities of laser light as well as corresponding filters were defined depending on experimental conditions. A "plate layout" (defining the wells of a multiwell plate) and a "sublayout" (defining the area insight the wells) where images should be acquired were defined.

All data from automated fluorescence imaging shown in this work have been acquired using the 20x air objective.

Data Analysis using the Acapella® software

An automated image analysis tool was developed in order to quantify cellular Venus fluorescence using the Acapella® software (PerkinElmer Cellular Technologies GmbH).

Here, nuclei (Supplementary Figure 12 A) and cell boundaries (Supplementary Figure 12 B) were automatically detected based on Draq5 signal. Based on this detection, an area for the quantification of mean cellular Venus fluorescence intensity was determined (Supplementary Figure 12 C). By setting an arbitrary threshold for the mean cellular Venus fluorescence intensity, cells were considered Venus-positive (Supplementary Figure 12 D) or Venus-negative (Supplementary Figure 12 E). As a final result, a table with high content data concerning different properties was obtained (Supplementary Figure 12 F).

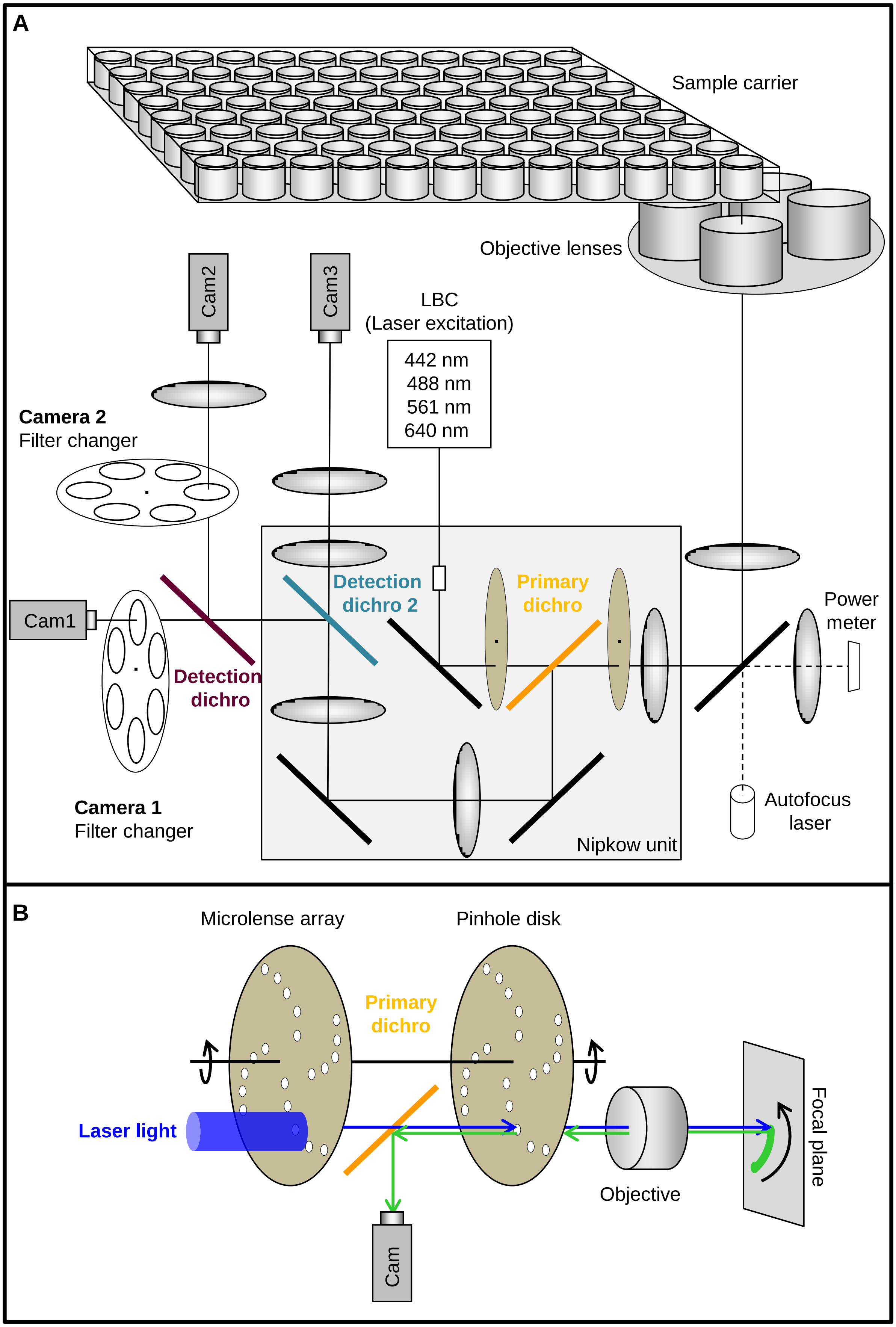
A
Sample carrier
Objective lenses
Cam2
Cam3
LBC
(Laser excitation)
442 nm
488 nm
561 nm
640 nm
Camera 2
Filter changer
Cam1
Detection dichro 2
Primary dichro
Power meter
Detection dichro
Camera 1
Filter changer
Autofocus laser
Nipkow unit
B
Microlense array
Pinhole disk
Primary dichro
Laser light
Objective
Focal plane
Cam

Supplementary Figure 11. Measurement setup of the Opera®

Schematic representation of the Opera® high-throughput confocal imaging platform and the Nipkow unit.

A) Laser light from four lasers (442 nm, 488 nm, 561 nm, and 640 nm) can be focused on the sample carrier via dichroic mirrors. Emitted photons pass the objective retrogradely and are guided via dichroic mirrors and a Nipkow unit to be detected separately using three different cameras.

B) The Nipkow disk inside the Nipkow unit consists of small holes arranged in an arched manner. Inside the Nipkow unit laser light is focused by microlenses in a disk with similar arrangement through the Nipkow disk and the objective on the sample. Emitted fluorescence is guided retrogradely through the Nipkow disk where the single holes decrease background fluorescence by excluding signals from above or below the focus plane. Due to the rotation of the Nipkow disk an arched image is recorded for each hole. The images of the different holes will finally add up to a complete image.

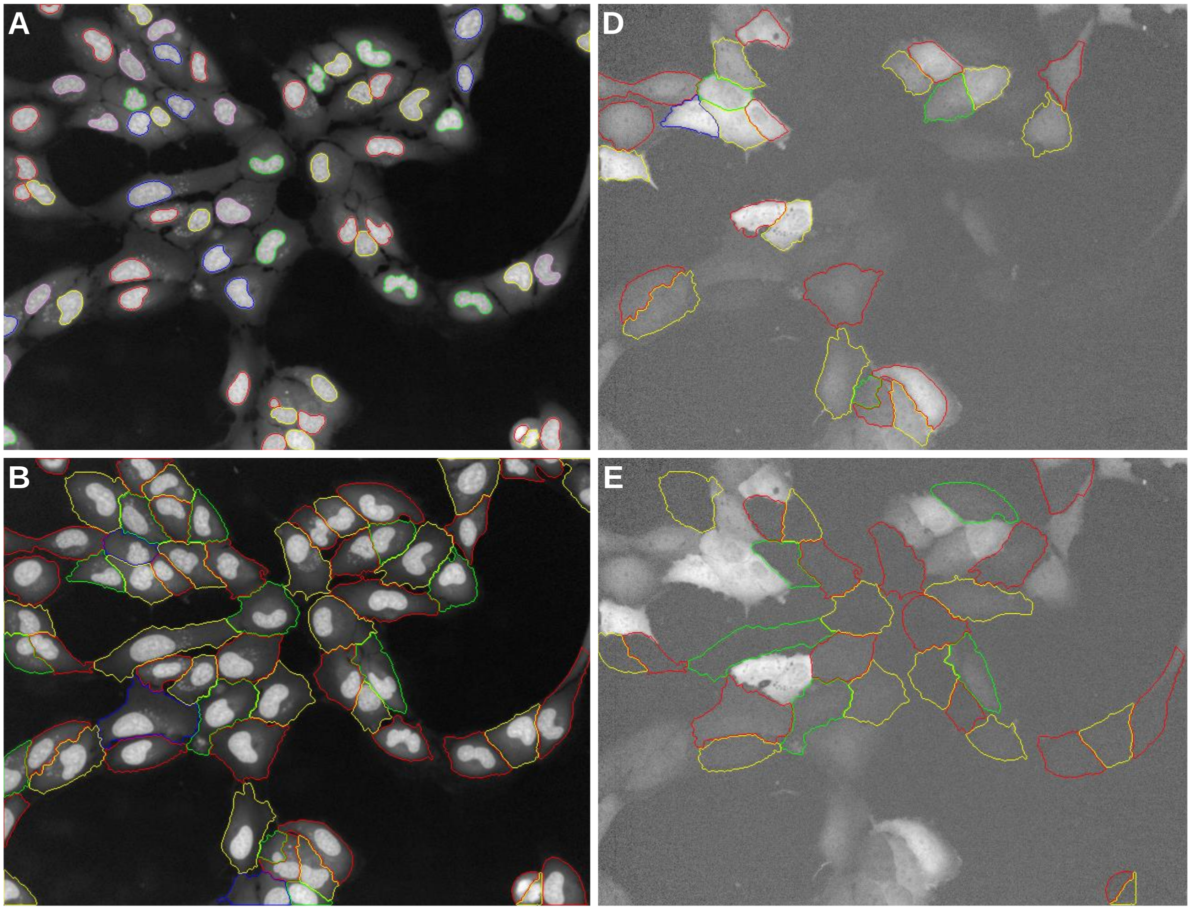

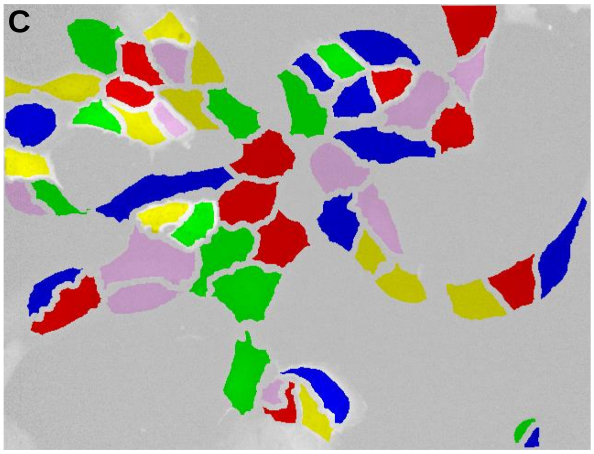

F

Analyzed image fields	1
Number of cells	54
Mean cellullar venus fluorescence intensity	70.5818
Median cellullar venus fluorescence intensity	45.2011
Stddev venus fluorescence intensity	47.2796
Number of venus-positive cells	25
Percentage of venus-positive cells	46.2963

Supplementary Figure 12. Acapella® data analysis

Overview of the automated image analysis using the Acapella® software.

A) Detection of nuclei based on Draq5 fluorescence.
B) Determination of cell boundaries based on Draq5 fluorescence.
C) Definition of the area to quantify mean cellular Venus fluorescence.
D) Cells considered Venus-positive based on an arbitrarily set threshold for mean cellular Venus fluorescence.
E) Cells considered Venus-negative based on an arbitrarily set threshold for mean cellular Venus fluorescence.
F) Final output table showing different sample properties.
